# Exploiting the Specificity of CRISPR/Cas System for Nucleic Acids Amplification-Free Disease Diagnostics in the Point-of-Care

**DOI:** 10.1021/cbe.3c00112

**Published:** 2024-01-09

**Authors:** Bong Jing Yee, Nurul Ajeerah Ali, Noor Faizah binti Mohd-Naim, Minhaz Uddin Ahmed

**Affiliations:** † Biosensors and Nanobiotechnology Laboratory, Integrated Science Building, Faculty of Science, Universiti Brunei Darussalam, Gadong 1410, Brunei Darussalam; ‡ PAPRSB Institute of Health Science, Universiti Brunei Darussalam, Gadong 1410, Brunei Darussalam

**Keywords:** CRISPR/Cas, Cas12, Cas13, Amplification-free, Diagnostics, Disease, Biosensors, Point-of-care

## Abstract

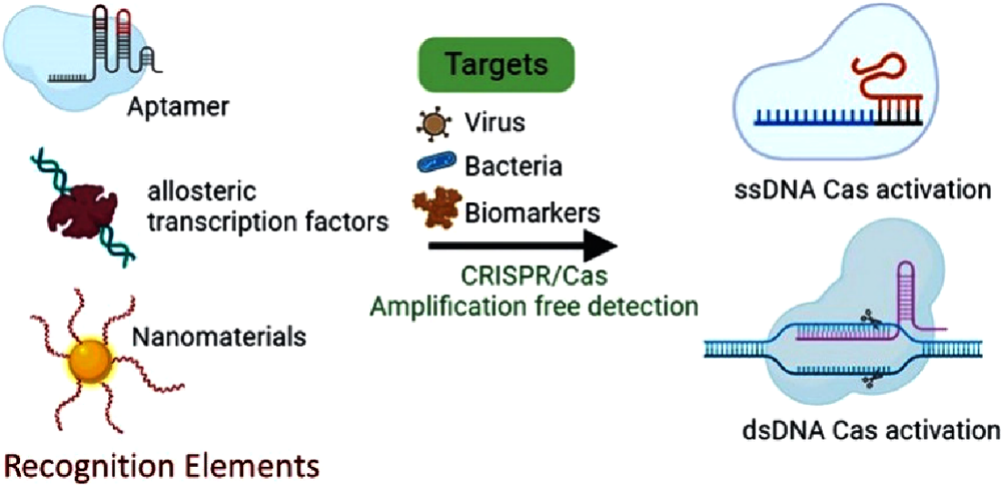

Rapid and reliable molecular diagnostics employing target nucleic acids and small biomarkers are crucial strategies required for the precise detection of numerous diseases. Although diagnoses based on nucleic acid recognition are some of the most efficient and precise procedures, these tests often require expensive equipment and skilled professionals. Recent advancements in diagnostic innovations, particularly those based on clustered regularly interspaced short palindromic repeats (CRISPR), aim to provide thorough screening at homes, in clinics, and in the field. In comparison to traditional molecular techniques like PCR, CRISPR/Cas-based detection, using the single-stranded nucleic acid trans-cleavage abilities of Cas12 or Cas13, shows significant potential as a molecular diagnostic tool. It offers benefits such as attomolar-level sensitivity, single-base precision, and rapid turnover rates. Both Cas enzymes demonstrate exceptional specificity and sensitivity, holding substantial promise in disease diagnostics and beyond. Consequently, various amplification-free CRISPR/Cas-based detection methods have emerged, aiming to maintain sensitivity despite the absence of pre-amplification. This allows for the detection of non-nucleic acid targets and facilitates integration into point-of-care settings. This Review highlights current advances in amplification-free CRISPR/Cas detection systems in disease diagnostics and investigates their utility in point-of-care settings. Furthermore, the mechanisms of alternative CRISPR-based amplification-free detection of other small molecules, aside from nucleic acids, for disease diagnosis will also be briefly discussed.

## Introduction

The ability to diagnose an illness quickly and accurately is critical for successful treatment and the prevention of long-term consequences.^1^ In the words of Alain Mérieux, “Without diagnostics, medicine is blind.” Providing adequate and timely treatment for diseases is impossible without a thorough diagnosis. The creation of novel diagnostic biosensors has expanded dramatically in recent years, as it has been demonstrated that early detection may radically alter the progression of a disease. The availability of biosensors for the most prevalent illnesses might save countless lives in developing countries. Unfortunately, some diagnostic methods are still too expensive, requiring equipped facilities and qualified personnel to perform them. These challenges are likely the most significant barriers to their adoption in these locations. According to the World Health Organization, diagnostics for developing nations should be ASSURED: affordable, sensitive, specific, user-friendly, rapid and robust, equipment-free, and deliverable to end-users with minimal trade-offs between affordability and accuracy.^2^

Nucleic acid testing based on polymerase chain reaction (PCR) and next-generation sequencing has been the gold standard for diagnosing infectious diseases and cancer. However, the incredible complexity and cost prevent them from being routinely used at the point-of-care.^3^ Isothermal nucleic acid testing methods such as Loop-mediated Isothermal Amplification (LAMP) and Recombinase Polymerase Amplification (RPA) have low equipment demands, and target nucleic acids are amplified linearly at a fixed temperature. However, nonspecific amplification products are commonly produced, resulting in reduced detection accuracy.^4^ In recent years, a revolutionary nucleic acid detection technique based on clustered regularly interspaced short palindromic repeats (CRISPR) and its associated protein systems, Cas, has provided extraordinary possibilities in this area.^5^

The CRISPR/Cas system is an adaptive immune system comprising RNA-guided endonucleases to defend its hosts from invading bacteriophages and other mobile genetic elements (MGEs).^6−8^ Researchers in various biotechnology disciplines, including infectious disease and cancer detection, have become interested in the CRISPR/Cas system due to its broad set of Cas proteins and genomic loci architecture since its initial discovery as a versatile genetic editing tool.^9^ CRISPR/Cas systems can be engineered for diagnostic applications to provide molecular detection platforms appropriate for point-of-care use in order to promote fair and equal access to disease diagnostics. In this Review, we highlighted the latest improvements in using CRISPR/Cas systems in amplification-free biosensing platforms to identify, diagnose, and characterize various diseases via specific recognition of nucleic acids and various small molecules. Furthermore, we discuss possible applications of CRISPR/Cas-based diagnostic tools intended to fabricate simple, low-cost, and portable point-of-care devices.

## CRISPR/Cas9-Based Biosensors for Disease Diagnostics

### Viral RNA Detection

**Figure 1:**
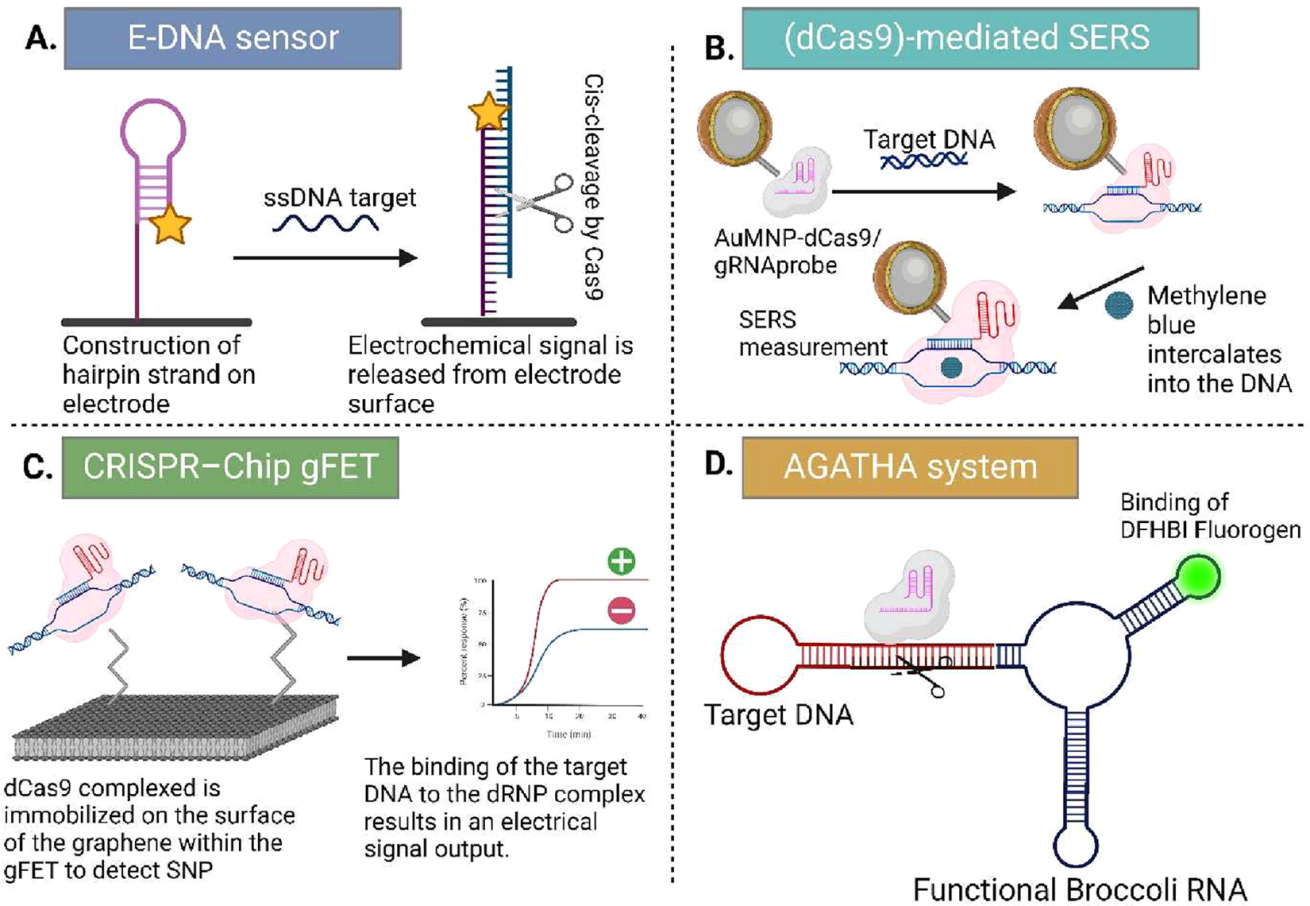
Schematic illustration of various Cas9 biosensors. (A) Illustration of the E-DNA sensor using electrochemical signals with the MB tag via ssDNA. Created with BioRender.com with permission from ref (10). Copyright 2022 Elsevier Inc. (B) Principal schematic of the ()-mediated biosensor illustrating the interaction of Au MNP-dCas9/gRNA probes with MDR bacterial genes to detect superbugs. Following Raman dye incorporation and magnetic separation, the multi-drug-resistant genes captured by the Au MNP-dCas9/gRNA probes produced strong SERS signals.^12^ Created with BioRender.com. (C) Schematic diagram of the RNA-guided dCas9 complex on the graphene surface within the CRISPR-Chip gFET, which can bind, enrich, and detect the target DNA leading to an output of an electrical signal. Created with BioRender.com with permission from ref (13). Copyright 2019 The Authors. (D) The developed AGATHA system involves AGATHA DNA expressing an inactive Broccoli RNA aptamer constrained by a 3′-end secondary structure (anti-Broccoli tail). When detecting the target RNA, activated Cas9 halts the transcription of the 3′-end secondary structure, enabling uninterrupted transcription of a functional aptamer. This functional aptamer can bind to DFHBI, resulting in an amplified fluorescent output signal.^14^ Created with BioRender.com.

The Cas9 effector’s cis-cleavage and binding activities are harnessed for nucleic acid recognition. In detection methods involving cleavage activity, the CRISPR/Cas9 system is employed to cleave target double-stranded DNA (dsDNA) labeled with electrochemical tags. This cleavage generates signals indicating the presence of specific targets. Xu et al. devised an electrochemical biosensor (E-DNA sensor) for detecting Parvovirus B19 (PB-19), a virus linked to erythema infectiosum in infants and pregnant women. This sensor operates by utilizing the cleavage activity of the Cas9 effector.^10^ See Figure 1. This process occurs when a dsDNA is formed at the electrode surface by the target ssDNA hybridizing with a reporter ssDNA conjugated with methylene blue (MB). Following the dsDNA’s reconfiguration, the CRISPR/Cas9 system cuts it, resulting in an electrochemical signal shift. A dynamic range spanning seven orders of magnitude was achieved, with a limit of detection (LOD) of 100 fM.

Xu et al.^10^ investigated enhancing the functionality of the E-DNA sensor by exploring a different class of CRISPR/Cas12a system. Unlike CRISPR-Cas9, Cas12a operates through double-stranded DNA (dsDNA) cleavage guided by its specific crRNA, making it a simpler system than Cas9. Cas12a identifies the complementary target via PAM recognition and crRNA matching, conducting targeted DNA *cis*-cleavage and triggering an additional single-stranded deoxyribonuclease activity known as *trans*-cleavage.

By applying both *cis* and *trans* cleavage activities of Cas12a to the E-DNA sensor, the study aimed to enhance its detection limit and dynamic detection range. Experimentally, they determined a detection limit of 10 fM for both buffer and pooled human blood testing with an average standard error of 2.8%. This finding demonstrates the robust and consistent detection capability of Cas12a.^11^

## CRISPR/Cas13-Based Biosensors for Disease Diagnostics

RNAs play crucial regulatory roles in tissue proliferation and disease development. Rapid and dependable approaches for ultrasensitive RNA detection and quantification can help us comprehend gene expression levels and provide insight into their involvement in cellular function and malfunction. There is an urgent need for high-sensitivity, affordable RNA diagnostics because precise and appropriate detection enables better diagnosis and treatment of infectious diseases and early identification of cancers.

Existing methods for RNA detection in clinical samples based on real-time quantitative reverse transcription PCR (qRT-PCR) offer excellent sensitivity but are too complicated to adopt for point-of-care. Despite the fact that there are additional isothermal-based RNA detection methods available, both PCR and isothermal amplification methods require the use of reverse transcriptase enzymes and custom primers, which are time-consuming processes.^15,16^ The programmable, RNA-guided detection of RNA sequences by CRISPR/Cas13 proteins makes them a more attractive alternative than PCR- and isothermal-based techniques.^17−19^

Cas13 protein lacks the HNH and RuvC domains in Cas9 and Cas12 that are necessary for targeting DNA.^20^ They can form a nuclease-inactive RNP complex: a crRNA that contains a programmable spacer region adjacent to a PFS. When the RNP binds to the complementary sequence found on the target RNA, the higher eukaryotes and prokaryotes nucleotide-binding domain (HEPN) motif becomes activated to cleave neighboring ssRNAs in a random manner.^21,22^ Cas13a, Cas13b, Cas13c, and Cas13d are the subtypes of CRISPR/Cas13 identified so far.^23^

### Viral RNA Detection

SATORI is a dependable, quick, and robust application for detecting ssRNA at a single-molecule level.^24^ Compared with other ssRNA detection, it offers significant technological benefits. The breakthrough couples CRISPR/Cas13 with microchamber technology to create a platform capable of precise and rapid detection of target ssRNA in the femtomolar range, making it less susceptible to amplification errors like PCR.^25^ The SATOR platform integrates Cas13a-mediated RNA detection with a microchamber array device, featuring over 1 million femtoliter microchambers. This setup enables extensive and simultaneous monitoring of chemical reactions at the single-molecule level. In a proof-of-concept experiment, *Leptotrichia wadei* Cas13a (LwaCas13a) and CRISPR RNA (crRNA) were employed to detect a target single-stranded guided RNA (tgRNA) in the microchamber device. The LwaCas13a protein, preassembled with a crRNA complementary to the 120-nucleotide tgRNA, was introduced into a sample solution containing tgRNA and fluorophore quencher (FQ)-labeled RNA reporters. Upon introduction of the mixture into the microchamber device and sealing it, fluorescence microscopy illustrated an enhanced fluorescence signal throughout the microchamber array. This finding directly supports the effective identification of tgRNA by LwaCas13a–crRNA complexes and their subsequent cleavage of FQ reporters within the microchambers. Their resistance to contaminants, such as saliva, further eliminates the need for RNA purification and other sample pretreatment procedures, which is beneficial for immediate clinical sample testing. Its rapid detection of 5 min is comparable to other systems that require 30 min.^26^ SATORI has the potential to be a robust and effective viral RNA recognition tool for not only the SARS-CoV-2 N-gene but also other RNA viruses like HIV, Zika, Ebola, and Influenza.^25^ SATORI will also permit amplification-free identification of double-stranded DNA (dsDNA) when paired with Cas12a, which would help to identify either viral DNA or circulating tumor DNA (ctDNA) from tumor cells. The selectivity study of SATORI revealed that mismatches between crRNA and target ssRNA (tgRNA) lowered the quantity of active *Leptotrichia wadei* Cas13a (LwaCas13a) −crRNA–tgRNA molecules but did not affect the trans-cleavage activity on LwaCas13a. However, the results depended on the location of the mismatch, similar to prior research observations using SHERLOCK.^27^

Previously, Kellner et al.^28^ developed a CRISPR-based diagnostic tool that integrates nucleic acid preamplification using CRISPR/Cas enzymology. SHERLOCK (Specialized High-Sensitivity Enzymatic Reporter Unlocking) enables multiplexed, mobile, and ultrasensitive SARS-CoV-2 RNA detection using Cas13 proteins requiring reverse transcription and pre-amplification processes that take around an hour to perform.^29−31^ SHERLOCK is versatile, supporting different detection methods, such as fluorescence or lateral flow, depending on the chosen reporter molecule. While either Cas12 or Cas13 enzymes can be utilized for detection, this protocol emphasizes Cas13. For Cas13 activation, a T7 RNA polymerase promoter is introduced during pre-amplification, and a T7 RNA polymerase is required in the detection reaction to generate RNA. Fluorescence detection can occur either as an end point or in real time using a plate reader or compatible fluorometer. On the other hand, lateral flow detection is an end point assay, involving exposure of lateral flow strips to the reaction mixture after incubation. SHERLOCK has now evolved into SHINE (Streamlined Highlighting of Infections to Navigate Epidemics), a diagnostic test that eliminates the requirement for nucleic acid extractions or any specialized equipment, exhibiting comparable ease of use as other nucleic acid diagnostics.^32^ Paper-based lateral flow strips, ideal for bedside testing, are utilized in SHINE to maintain simple and intuitive data interpretation. The compatibility with the lateral flow platform might be critical in emergencies, providing precision and speed in presenting results quickly.^29^ The lateral flow readout is advantageous in terms of minimal equipment requirements, only requiring a heat block. However, they require extended incubation periods to detect samples with lower viral titers. This method is less conducive to testing numerous samples concurrently and poses a risk of potential sample cross-contamination due to the manual insertion of lateral flow strips into opened tubes for each sample.^33−35^

## CRISPR/Cas12-Based Biosensors for Disease Diagnostics

CRISPR/Cas12 proteins, as mentioned earlier, are RNA-guided enzymes that recognize complementary double-stranded DNA (dsDNA) or single-stranded DNA (ssDNA) containing T-rich PAM sequences. Cas12 effectors cleave target DNA mediated by a single RuvC domain and can subsequently activate the trans-cleavage activity on nonspecific ssDNA.^36^ The type V CRISPR/Cas systems have at least eight subtypes (Cas12a, -b, -c, -d, -e, -f, -g, -h, and -i), but only three are frequently used for CRISPR/Cas12-based diagnostic approaches.

Cas12a and Cas12b are among the most common subtypes. Cas12a is simpler to employ, unlike Cas12b, which requires the presence of tracrRNA to achieve interference.^37^ Cas12b has not yet been extensively studied in diagnostics due to the requirement for higher temperatures (at 48°C); however, efforts have been made to engineer the enzyme for operation at lower temperatures.^38^ The high efficiency trans-cleavage activity (around 1250 rotations per second) and crRNA-dependent sequence specificity exhibited by Cas12a have been employed to detect nucleic acids with excellent specificity, sensitivity, and speed.^39^ Specifically, Cas12a endonucleases requiring only crRNA as their guide RNA can recognize T-rich PAM sequences to generate dsDNA breaks with staggered 5′ ends.^36^

Another subtype, Cas14 (also known as Cas12f), is becoming increasingly common in diagnostics because it requires complete complementarity in the seed region of sgRNA, which is necessary for achieving single nucleotide specificity.^40^ They exhibit a miniaturized structure comprising 400–700 amino acids, about half of the size of Cas9 and Cas12. Cas14 has been re-classified into the Cas12 family due to its similarity with Cas12, which depends on T-rich PAM and utilizes dsDNA or ssDNA as its target.^41,42^ Cas14 demonstrated excellent detection and discrimination between identical ssDNA sequences with one nucleotide variation comparable to Cas12a, even in the presence of a PAM sequence.^43^ We can expect Cas14 to be widely repurposed for amplification-free diagnostic applications to identify ssDNA SNPs without needing a PAM sequence due to their increased specificity and convenience.

### Viral DNA Detection

Noble metal nanoparticles are intensively characterized nanomaterials extensively employed in the development of techniques for diagnostic purposes, imaging, drug administration, and therapies^44^ due to fewer adverse effects and greater patient compliance.^45^ They exhibit a Raman enhancement phenomenon known as surface-enhanced Raman scattering (SERS), an alternative highly sensitive sensing technology for fluorescent transducer systems that could be implemented in CRISPR/Cas-based biosensors.^46^

Early detection of viral nucleic acids can be crucial in disease prevention, especially for DNA viruses like Hepatitis B virus (HBV), where the detection of DNA genomic material can be specifically recognized by reprogramming CRISPR/Cas12a. In combination with novel gold nanoparticles conjugated to 4-ATP via nonspecific ssDNA as a Raman probe, Du et al. established a CRISPR-SERS assay targeting HBV DNA.^47^ The Raman reporter molecules can amplify the SERS signal without the need for prior amplification. However, further optimization is needed to enhance signal strength without compromising detection sensitivity and portability.

The SERS-based CRISPR/Cas12a diagnostic assay (S-CRISPR) can identify the cDNA of SARS-CoV-2 N-gene RNA after reverse transcription with high specificity and sensitivity as low as 1 fM without amplification using a portable Raman plate reader. As reported by Liang and colleagues,^48^ a silver nanoparticle solution mixed with 4-ATP (AgNP@4ATP) was employed as a Raman tag, linked to magnetic beads (MB) via ssDNA to form the SERS probe, which is cleaved by activated Cas12a enzymes. The S-CRISPR assay is easy-to-use compared to amplification-based CRISPR assays and conventional RT-qPCR, taking only 30–40 min to complete. The short duration spent developing and verifying the assays demonstrates that such tests could rapidly detect diseases caused by the affected evolving pathogens. Further studies may also be performed to test the utility of this assay for detecting DNA from other infectious pathogens. However, replacing Cas12a with Cas13 in this assay may alleviate the need for an additional reverse transcription step on viral RNA samples, further simplifying the assay and reducing the test duration for use in point-of-care diagnostics.

**Figure 2:**
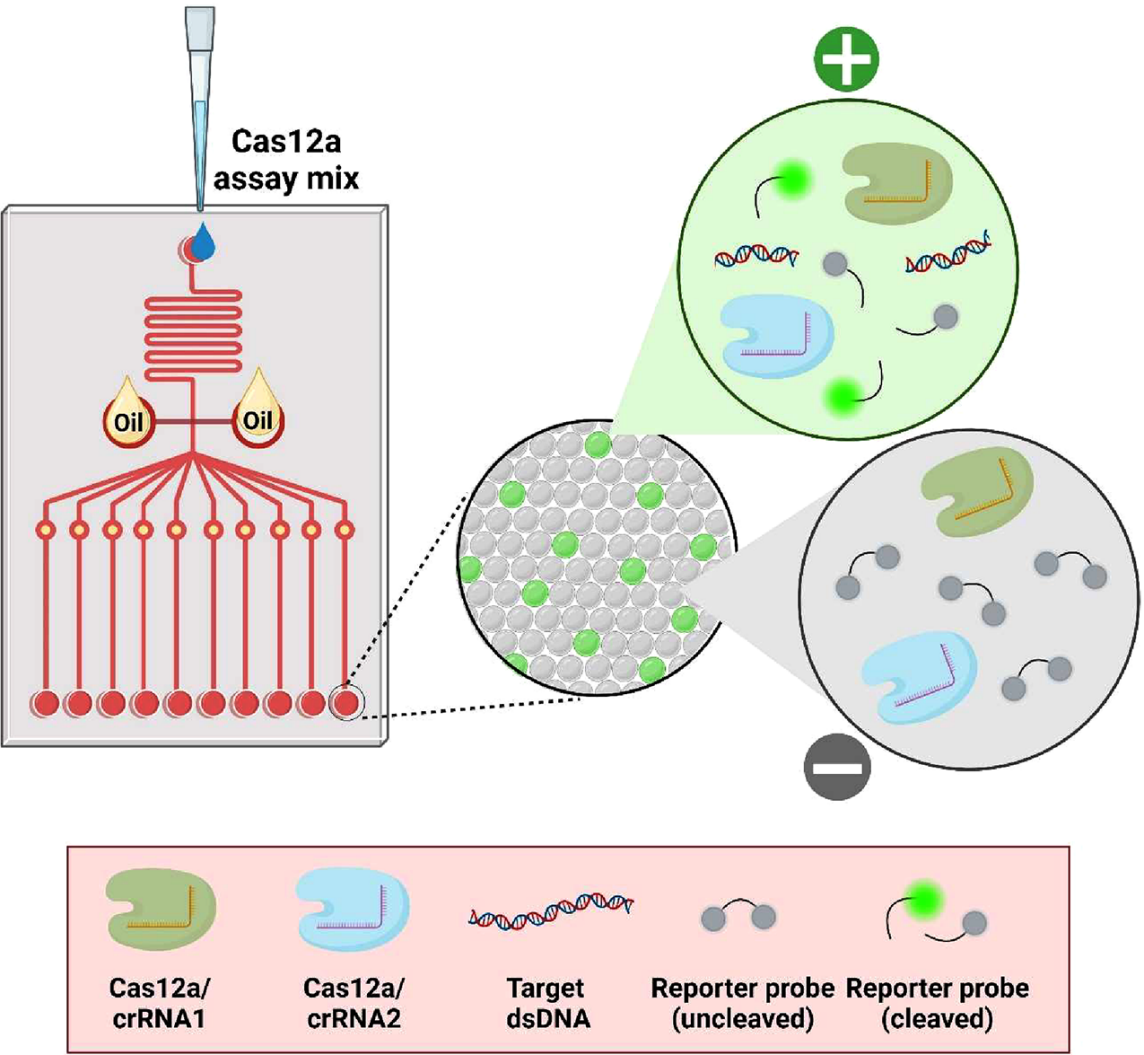
Illustration of a microfluidic droplet chip with sample input and oil inlets designed to generate a highly homogeneous layer of a droplet array using negative pressure. The assay includes a positive control of Cas12a, two crRNAs, a fluorescent probe, and target DNA, without a negative control. The droplet Cas12a assay demonstrates the formation of highly homogeneous microdroplets.^49^ Created with BioRender.com.

In the same year, Yue et al.^49^ developed a CRISPR/Cas12a droplet assay for digital DNA absolute quantification at the single-molecule level by employing dual-crRNA targeting using a microfluidic droplet chip (Figure 2). Because the trans-cleavage efficiency of Cas12a is half of the Cas13 system, this allows for the direct measurement of the target sequence in the picomolar range.^50^ Maximizing the efficiency of the reaction parameters, namely optimizing buffer and increasing reaction temperature, can considerably improve the assay by 50-fold compared to existing Cas12a assays to directly detect DNA concentrations as low as 100 fM.^51^ The utility of this assay was tested using DNA from various disease-causing viruses, such as African swine fever virus (ASFV), Epstein–Barr virus (EBV), and HBV in clinical serum samples by using crRNA flexible programmability. Without the need for target amplification, this test can correctly identify all positive and negative samples due to its high specificity, and the results were fully compatible with qPCR. The droplet Cas12a assay beats qPCR in direct DNA measurements without requiring performance standards. In contrast to digital PCR, the droplet Cas12a assay enables rapid detection of single unamplified DNA molecules, eliminating any amplification mistakes produced by enzymes and starting primers, making it more accurate for adoption at the point-of-care.

## Non-Nucleic Acid (NNA) Target Detection

**Figure 3:**
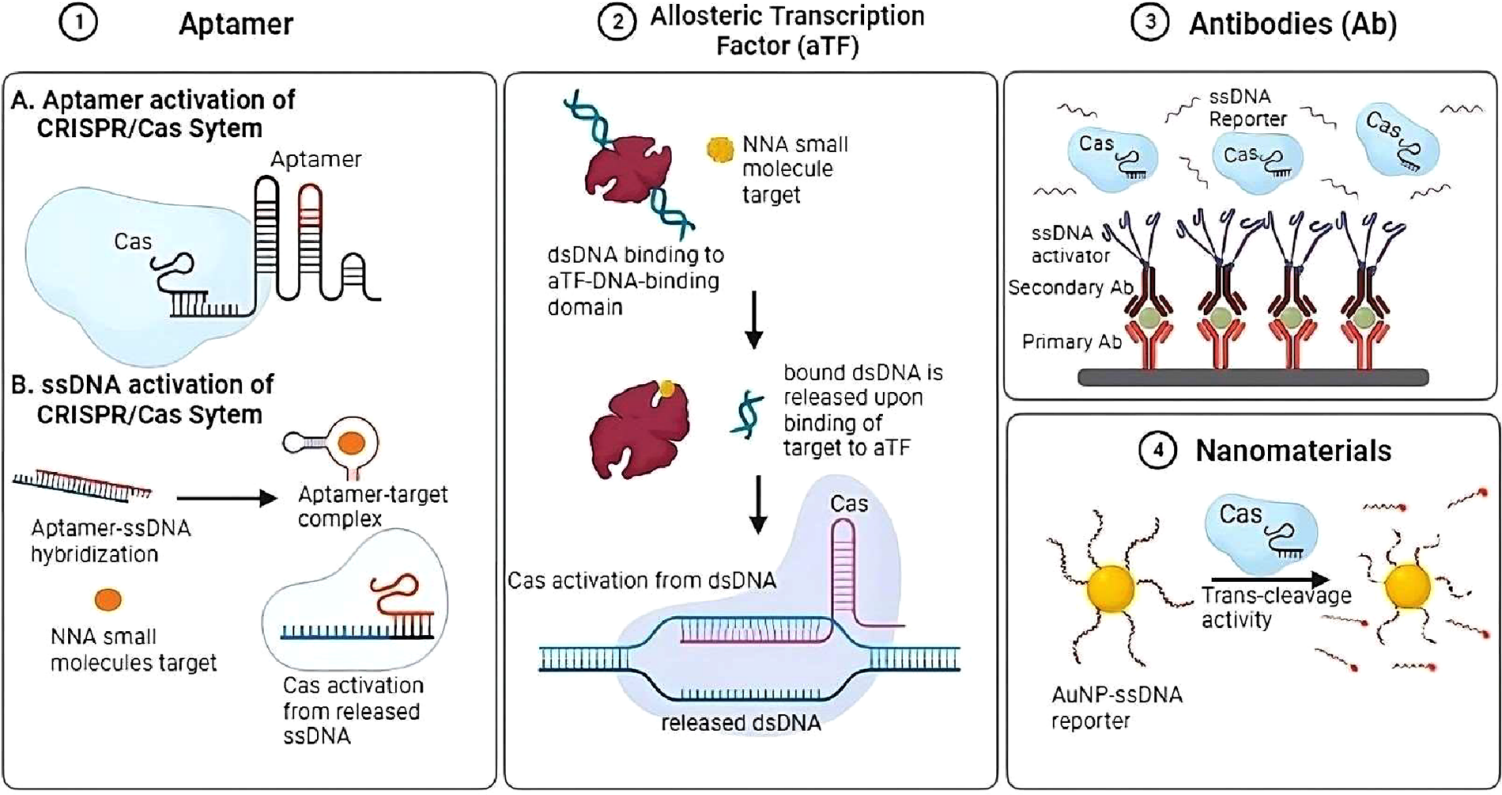
Schematic illustrations depict the incorporation of biological components for NNA target detection using a CRISPR/Cas system amplification-free approach. Created with BioRender.com.

CRISPR/Cas systems are widely used to identify nucleic acids, but their applicability in identifying NNA targets has been constrained for a while. Clinically relevant NNA targets can be detected indirectly using signal-enhancing CRISPR/Cas12 technologies in conjunction with commonly used recognition elements such as aptamers,^52^ antibodies, and allosteric transcription factors (aTFs). This approach overcomes the need for amplification processes. NNAs bind selectively to the recognition elements, releasing specific nucleic acid sequences that activate the trans-cleavage activities of Cas12 enzymes on probes (Figure 3). Other emerging recognition elements, such as DNAzymes, have also been noted for their use in NNA target detection.^53,54^

Nanomaterials, defined as materials with at least one dimension ranging from 1 to 100 nm, have emerged as versatile tools in biosensing applications, owing to their unique optical, electrical, and thermal properties.^55^ Within the realm of CRISPR/Cas-based biosensing approaches, nanomaterials serve a multitude of functions, including recognition elements specifically targeting substances through functional groups or intermolecular interactions such as hydrogen bonds, electrostatic forces, and π–π interactions. They also act as carriers, coating biomolecules like proteins and nucleic acids, as well as separators, utilizing magnetic nanomaterials to facilitate isolation. Furthermore, nanomaterials can function as quenchers, reducing the fluorescence of molecules or luminescent groups through mechanisms such as fluorescence resonance energy transfer (FRET) and inner filter effect (IFE). Additionally, they can act as catalysts, exhibiting peroxidase or oxidase properties, as conductors, facilitating electron transfer on electrode surfaces through conductive nanomaterials, and as reporters, possessing fluorescent, Raman, or electrochemical properties. Importantly, in practical applications, nanomaterials often fulfill multiple roles through integration rather than functioning in isolation, highlighting their versatility and adaptability in biosensing applications.^56^

The use of nanostructured materials in biosensing technologies with transducers for signal amplification in immunosensors has also resulted in a number of appealing platforms. Over time, significant advances in immunosensors have been reported, including a larger surface area, functional interface for biomolecule immobilization, adsorption capabilities, electron-transfer kinetics, and spatial arrangement. They also have excellent optical, electrical, and molecular characteristics, as well as desirable functionality. Furthermore, the increased physical features of nanomaterials such as shape, structural sizes, and configurations might improve antigen-antibody binding activities, enhancing immunoassay sensitivity.^57−60^ Therefore, rebuilding immunosensors by employing nanomaterials and nanocomposites can be an essential method for overcoming the mentioned limitations.

Nanomaterials have shown great potential in terms of transforming POC diagnostics and healthcare. They have unique features due to their nanoscale size, making them appropriate for a variety of applications in POC healthcare. Lab-on-a-chip (LOC) technologies have sparked substantial scientific interest due to their capacity to integrate many sophisticated analytical procedures onto a single chip,.^61^ Most large-scale laboratory procedures may be reduced to small chips by using such technologies. Nanomaterials are essential components of miniaturized lab-on-a-chip systems capable of performing several diagnostic procedures on a single platform. These devices use nanomaterial-based sensors and microfluidics to analyze biomarkers for diseases such as heart disease, diabetes, and infectious diseases. The incorporation of nanomaterials into CRISPR-based biosensors may increase the detection capability, leading to the development of a new class of POC nanobiosensors for quick and sensitive diagnostics.

## Future Outlook and Conclusions

Although existing sensing technologies are capable of detecting biomarkers with high accuracy and specificity, they are considered insufficient in meeting the three intrinsic limits of biomarker determination: fast, low-concentration, and low-cost measurement.^62^ Point-of-care biosensor technologies essentially eliminate these disadvantages and provide multiple advantages such as faster response times, simpler handling, affordability, small sample volume, and high sensitivity.^63^ POC testing needs to be both cost-effective and medically effective, as these are the two factors that will ultimately improve both the medical system and the economy.

Point-of-care (POC) testing is one of the most groundbreaking medical developments, allowing for the quick capture of diagnostic test data in real time. Compared to having test results delivered by a pathology laboratory, access and immediacy of results utilizing POCT have led to the same or better medication adherence, in addition to the apparent diagnostic benefits.^64^ These devices have become increasingly common, with intriguing implications in the domains of personalized medicine due to their ability to identify various biomarker-based disorders. They were marketed for diagnosing and monitoring a variety of conditions, including cancer, diabetes, cardiovascular disease, and infectious diseases.^65,66^

### Wearable POC Sensors

Wearable chemical sensors present an enticing prospect for simultaneous monitoring of a broad spectrum of molecular signatures, laying the groundwork for comprehensive multi-omics analysis. The ability to continuously monitor and discern temporal trends holds significant promise in the exploration of biomarkers characterized by rapid temporal dynamics.^67^ Wearable biosensors might be used to monitor cardiac, epilepsy, or other acute disorders with the goal of understanding the physiological changes in the body prior to such occurrences. Currently, there is no equipment or tests that predict life-threatening occurrences like heart attacks, epilepsy episodes, or heat strokes. Monitoring various biomolecules in real time throughout an individual’s regular activities might reveal crucial information about the chemical signature of aberrant health issues. This ability allows for the rapid screening of molecular signatures, which, when combined with data-mining techniques, results in a more successful biomarker discovery process that cannot be achieved with any other analytical method.^68^ Wearable sensors capable of detecting DNA, RNA molecules, fragments, hormones, proteins, and whole organisms, including viruses, will be crucial for public health monitoring and surveillance.

In a study by Yang et al.,^69^ a significant advancement was proposed for the precise, continuous, and real-time assessment of unamplified target DNA. The researchers employed reverse iontophoresis along with CRISPR-Cas9-activated graphene biointerfaces on conductive microneedles (MNs) to monitor nucleic acids in real time. This CRISPR-based wearable device was applied for monitoring Epstein–Barr virus, sepsis, and kidney transplant cell-free DNA (cfDNA), capitalizing on the combined effect of CRISPR-Cas9 and graphene biointerfaces. This method holds promise for the long-term real-time monitoring and early detection of diseases derived from cfDNA.

The proposed wearable platform consists of a functional flexible patch created through spray-printing and three-electrode conductive microneedles to enable real-time monitoring of target DNA. Initially, the polydimethylsiloxane (PDMS) membrane surface was modified with plasma to enhance hydrophilicity. This modified PDMS membrane was then coated with a hydrophilic layer. The percolating microstructure of PDMS would deform under bending, stretching, or twisting due to its soft properties and weak surface adherence. Carbon nanotubes (CNTs) were subsequently deposited on the modified PDMS sheet, forming a pattern for reverse iontophoresis to selectively segregate negatively charged substances, such as nucleic acids or ascorbate. Finally, a conductive CRISPR microneedle array was affixed to the anode side of the CNT pattern, serving as the working electrode for recording electrical signals.^69^

Although CRISPR-based sensing devices allow for the specific discrimination of nucleic acid signatures, it remains largely unexplored as an application for wearable sensors. Wearable detection of infection by external pathogens,^70^ localized cellular damage, or even cancer surveillance^67^ might be possible with nucleic acid sensors. Field-deployable platforms have been robustly developed using CRISPR proteins’ highly specific nucleic acid-targeting activity and distinct collateral cleavage ability. These platforms showcase detection sensitivity superior to conventional laboratory-based PCR methods, like reverse transcription (RT–PCR).^70,71^ Cas13a and Cas12a platforms have been designed for detecting RNA and DNA targets, respectively. The target-activated nonspecific nuclease activity allows for signal amplification and highly sensitive detection. Furthermore, CRISPR-based sensors may be readily customized by replacing the target-determining gRNA. CRISPR-based systems have only been employed in wearable sensors for virus detection and breath-based face mask detection.^67^

The discovery of the CRISPR/Cas immune system, as well as the characterization of Cas enzymes, has revolutionized gene editing and molecular diagnostics, resulting in an incredible number of rapidly expanding applications. Cas effectors have grown into useful biorecognition components employed in many biosensing systems because of their excellent selectivity and sensitivity toward nucleic acids. These fundamental properties may enable us to overcome several critical constraints in CRISPR-based POC devices, bringing their performance up to par with that of PCR-based procedures.^72^ However, with barely half a decade of study, the area of CRISPR sensing is still in its early stages and there are still many challenges to overcome, specifically in CRISPR-based POC applications.^73^

### Challenges and Potential Solutions of Applying CRISPR/Cas in POC

1. Sequence restrictions
  One common challenge in CRISPR sensing is the restricted sequences that CRISPR/Cas effector proteins can detect. Cas12a, for instance, has a lower mismatch tolerance and higher specificity than Cas9. When designing CRISPR studies for high specificity, careful consideration must be given to the effector protein and the detecting region on the target sequence to prevent non-specific but low background signals.
2. Standardization
  The key to successful POC CRISPR sensing lies in standardization. Both techniques and individual tests need to be standardized to ensure consistent results for every user. This is especially crucial for protein-based sensors. Factors such as salt levels, temperature, pH, and quantity of reaction inhibitors can interfere with gRNA and effector assembly for cleavage activity, leading to a shift in the produced signal. While challenges in consistency are common in POC testing, providing a test dilution series for on-site calibration or spiking samples with known levels can address these difficulties, albeit at the cost of some testing convenience.
3. Sample processing
  Minimizing the number of steps required to conduct an assay is crucial in developing a POC kit. Ideally, a kit for home-use diagnostics should deliver a straightforward sample to a diagnostic solution with minimal end-user handling. Despite advancements in both amplification-free and amplification-based detection, sample processing remains a barrier. Traditional sample processing procedures involve extensive liquid handling processes and a high reliance on specialized instrumentation, introducing complexity and potentially reducing recovery efficiency at the expense of sample purity.^74^
4. Quantitative analysis
  Quantitative analysis is essential for molecular diagnostics, particularly in diseases, where the amount of nucleic acids discovered in liquid biopsies varies with disease development. Quantification may be necessary for an accurate diagnosis. However, without an on-site standard, quantifying results becomes challenging.
5. Multiplexing
  Accurate genetic diagnostics requires the simultaneous detection of multiple targets, known as multiplexing. Achieving multiplexed sensing in a single reaction is challenging due to limits in signal-gathering approaches, interference between different detection phases, and potential cross-reactions compromising sensitivity and specificity. Non-specific cleavage of the same nucleotide detected during single-reaction multiplexing is a significant issue, leading to non-specific collateral cleavage of sequences and potential destruction of other targets.
6. Performing multistep assays in a single step
  The primary aim of CRISPR molecular diagnostics is the integration of sample processing, detection, and readout within a single assay. Simplifying diagnostic kit development involves enclosing chemicals within a compact device and streamlining the processing of these reagents. Different platforms such as tube-based devices, paper-based solutions, and microfluidics modules have been extensively researched, offering diverse sample-to-result solutions.

The ability of Class II CRISPR/Cas proteins to exhibit multigenome editing capabilities has paved the way for novel methods in diagnostics. Cas proteins demonstrate excellent specificity in target nucleic acid identification and binding when guided by crRNAs. The characterization of the trans-cleavage activities exhibited by Cas12 and Cas13 enhances detection sensitivity compared to conventional PCR methods, accelerating the advancement of CRISPR/Cas systems beyond nucleic acid detection. Combining CRISPR/Cas systems as a signal amplifier with recognition approaches will further expand the application of CRISPR/Cas diagnostics for clinically relevant NNA biomarkers, such as antibiotics and metabolic small molecules.^75^

When considered collectively, current amplification-free approaches employ various diagnostic concepts, offering varying levels of limit of detection (LOD) and operational ease. CRISPR/Cas methods exhibited higher precision compared with isothermal amplification techniques. Successful implementations of CRISPR/Cas systems without amplification steps have been demonstrated, with ongoing developments anticipated in the future. The elimination of amplification steps reduces the risk of aerosol contamination, which is particularly crucial in infectious disease diagnostics. Most existing CRISPR/Cas-based diagnostics face a significant limitation in relying on amplification methods for identifying targets below the femtomolar range.^76^ While amplification primers provide an additional level of specificity, this complicates the test, increases costs, and extends reaction times. Skipping the target nucleic acid preamplification step significantly decreases detection time and eliminates the need for specialized equipment, making the entire detection process more convenient and cost-effective.

In addition to the aforementioned approaches, several initiatives aim to enhance the detection sensitivity. Signal amplification procedures, particularly through nanotechnology, play a crucial role. The innovative incorporation of nanoparticles into analytical biosensors enables significantly lower detection limits and higher sensitivity.^77−79^ The flexibility of crRNA, combined with optimization into one-pot reactions and simple visualization, allows for the easy application of CRISPR/Cas-based amplification-free diagnostics across various uses. Machine learning-assisted design of enzymes, high dependence on Cas nuclease activity, and potential off-target effects can be addressed through various strategies.^80−82^ Despite achieving several milestones, widespread deployment is hindered by specialized instrument requirements and high detection costs, necessitating further solutions.^83^

Despite recent achievements, CRISPR/Cas diagnostics are rapidly advancing for point-of-care use, spanning from clinical settings to home applications. The integration of microfluidic technology with CRISPR/Cas biosensing eliminates bulky instruments and enhances reagent storage.^49^ CRISPR/Cas systems’ tolerance to contaminants allows streamlined “one-pot” reactions without rigorous sample purification.^25^ Microfluidic technology enables the simultaneous detection of multiple targets. Achieving single-molecule sensitivity involves combining CRISPR/Cas systems with advanced optical sensing technologies, such as super-resolution microscopy. With ongoing improvements in signal detection methods, simplified and automated biosensor platforms, including those employing naked-eye detection, lateral flow assays, and mobile phone measurements, are gaining preference.

Continued technological developments, driven by the demand for rapid point-of-care diagnostics, are expected to integrate amplification-free CRISPR/Cas systems into wearables and medical devices for the real-time quantification of clinical disease biomarkers. Patients can remotely assess their diagnostic results using mobile applications after being integrated with artificial intelligence technologies. The substantial refinement of CRISPR/Cas biosensors for point-of-care applications will enable their widespread use in various fields in the future, including but not limited to food safety, environmental monitoring, and disease detection.

## Supporting Information Available

Further information regarding CRISPR/Cas Diagnostic Toolbox, CRISPR/Cas9-based Biosensors for Disease Diagnostics, CRISPR/Cas13-based Biosensors for Disease Diagnostics, CRISPR/Cas12-based Biosensors for Disease Diagnostics, Multiple Targets Detection, CRISPR with Sequencing, Non-Nucleic Acid (NNA) Target Detection, Detection Read-out Methods, and Strategies to improve the amplification signal of CRISPR/Cas system (PDF)
