# Supplementary material for: Exploiting the Specificity of CRISPR/Cas System for Nucleic Acids Amplification-Free Disease Diagnostics in the Point-of-Care
Source: Chem Bio Eng. 2024 Jan 9;1(4):330–9. doi: 10.1021/cbe.3c00112 (PMC11835143; doi:10.1021/cbe.3c00112)
Supplement: Supplementary file 1 — be3c00112_si_001.pdf [file be3c00112_si_001.pdf]

Supplementary Information

Exploiting the Specificity of CRISPR/Cas System for Nucleic Acids Amplification-free Disease Diagnostics in the Point-of-Care

Bong Jing Yee[a], Nurul Ajeerah Ali[a], Noor Faizah binti Mohd-Naim,[a,b] and Minhaz Uddin Ahmed[a *]

[a] *Biosensors and Nanobiotechnology Laboratory, Integrated Science Building, Faculty of Science, Universiti Brunei Darussalam, Gadong, Brunei Darussalam;*

[b]*PAPRSB Institute of Health Science, Universiti Brunei Darussalam, Gadong, Brunei Darussalam*

[*]Corresponding author: minhaz.ahmed@ubd.edu.bn

CRISPR/Cas Diagnostic Toolbox

Mechanism of CRISPR/Cas

Cas proteins were initially believed to cleave foreign DNA, originating from bacteriophages, transposons, or plasmids, into fragments of approximately 20 bp in length. These fragments are integrated into contiguous stretches of nucleotides known as CRISPR arrays. This array is preceded by an AT-rich leader sequence and flanked by a set of *cas* genes encoding the Cas proteins [1,2]. The effector complex responsible for RNA-guided nucleic acid cleavage comprises a guide RNA (gRNA), also recognized as CRISPR RNA (crRNA), along with a set of Cas proteins. Within this complex, crRNAs direct a Cas nuclease by enabling the hybridization of the spacer region of the crRNA to a target sequence adjacent to a protospacer adjacent motif (PAM) or protospacer flanking sequence (PFS). This interaction ultimately triggers the cleavage of the target nucleic acid [3].

Classifications of CRISPR/Cas System

CRISPR/Cas can be divided into two classes (refer to Table S1). Class 1 CRISPR/Cas systems, for example, contain multiple Cas protein effector complexes, while class 2 systems have only one Cas multidomain effector protein [4,5]. To date, scientists have discovered six types of CRISPR/Cas proteins with at least 33 subclasses and several variants. Due to their distinct evolutionary history, Class I and Class II effector proteins are considered completely unrelated [6].

***Table S1**. Two classes of CRISPR/Cas Systems*

Classes	Subtypes	Cas Endonuclease	Target	Presence of tracRNA
**Class I**	Type I	Cas3	DNA	No
	Type III	Cas10	DNA/RNA	No
	Type IV	Csf1	-	-
**Class II**	Type II	Cas9; dCas9	DNA; -	Yes
	Type V	Cas12; Cas14	DNA; ssDNA	No
	Type VI	Cas13	RNA	No

CRISPR/Cas Systems for Disease Detection

The Class II CRISPR/Cas systems have been predominantly used in diagnostics to identify nucleic acids in a sensitive and specific manner, allowing for a diverse range of applications (Fig. 1).

Therefore, the focus of this review will be solely on utilising Class II CRISPR/Cas for disease diagnostics.

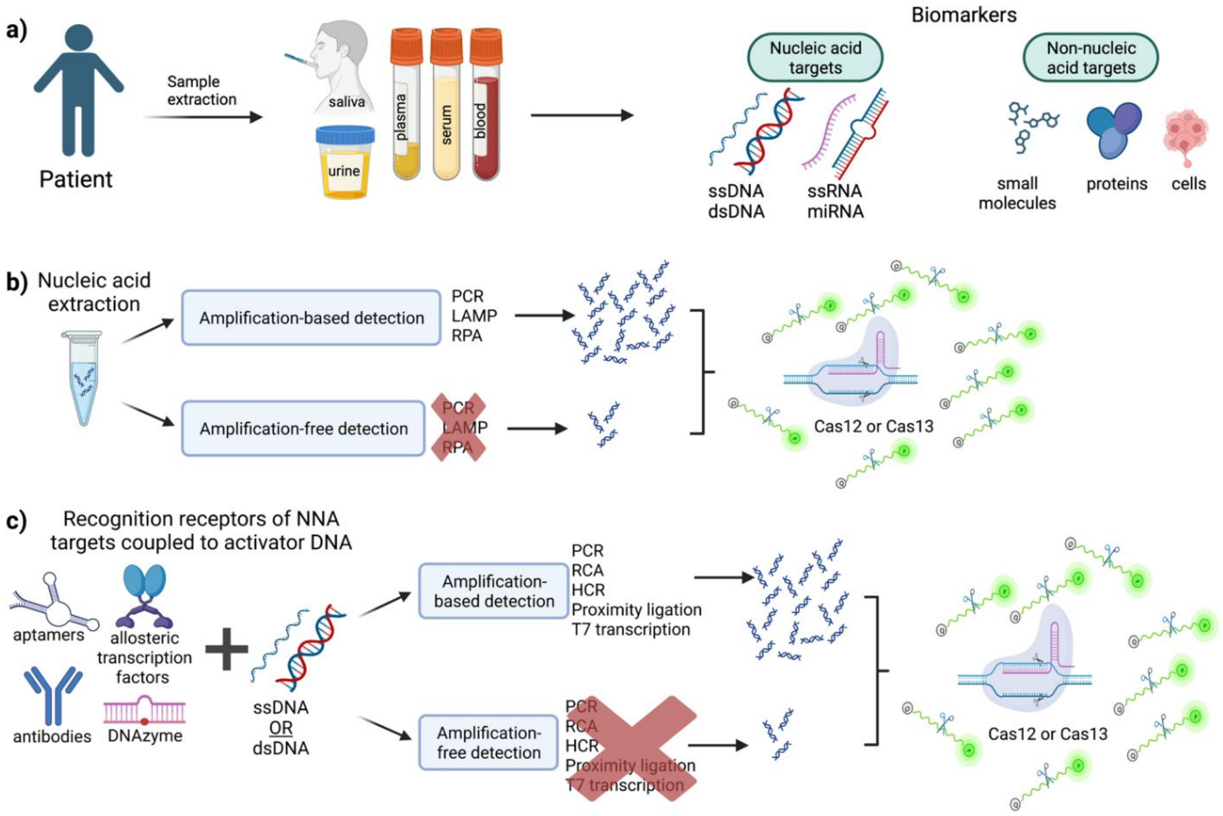

Figure S1. The Integration of CRISPR/Cas Platform in the Detection of Clinically Important Biomarkers. *a) a) Flow of sample extraction from patients to the identification of biomarkers. The two categories of biomarkers that can be identified in different types of samples are nucleic acid targets and non-nucleic acid (NNA) targets. b) Nucleic acid (DNA/RNA) diagnostic testing from patient samples. Purified nucleic acids were either subjected to amplification or no amplification steps. Target nucleic acids were then recognised by the CRISPR/Cas Class 2 system to initiate trans cleavage activity, which cleaves single-stranded DNA (ssDNA) conjugated to a fluorophore and quencher indiscriminately. c) Receptors that bind to NNA targets are used as a recognition element. The binding activity is then translated to quantitative signals via the amplification or amplification-free activation of the activator DNA. The nucleic acid is then used to activate the CRISPR/Cas system for the trans cleavage of the ssDNA-reporter molecules.*

Most early diagnostic innovations based on CRISPR/Cas systems relied on the Cas9 effector system, which could not generate a strong and specific signal in the presence of unamplified target dsDNA. Typically, these systems were paired with either pre-amplification [7], or post-amplification [8–11] steps of the target sequence using PCR or isothermal methods to achieve sensitivity levels up

to the attomolar range. Although the two stages are often executed separately, they can be merged in a single tube with rigorous amplification control and trans-cleavage parameters [12]. Nevertheless, these amplification strategies are time-consuming and pose an increased risk of contamination.

To address these constraints, various research groups have worked to develop CRISPR/Cas-based amplification-free techniques to to explore their potential use in disease detection. The detection of nucleic acids takes advantage of the intrinsic enzymatic activity provided by Cas12 and Cas13 effector proteins.

CRISPR/Cas9-based Biosensors for Disease Diagnostics

Viral RNA Detection

The type II CRISPR system utilizes trans-activating CRISPR RNA (tracrRNA) to form mature guide RNA (gRNA) in conjunction with precursor CRISPR RNA (pre-crRNA)[13]. TracrRNA plays a vital role in RNase III-dependent RNA processing, which leads to the creation of mature crRNA and the Cas9 ribonucleoprotein (RNP) complex. This process is essential for CRISPR functionality and involves intricate molecular interactions with the Cas9 protein. [14, 15]. Recent studies suggest the programmability of crRNA-tracrRNA pairings. Y. Liu et al. systematically investigated the programmability inherent in crRNA-tracrRNA pairs within the CRISPR/SpCas9 system. Their objective was to create programmable devices. The remarkable programmability observed in these pairings paves the way for engineering and utilizing the CRISPR/Cas9 tool in novel ways, enabling the development of orthogonal mechanisms and logic gates based on this system's functionality.

Moreover, Y. Liu et al. demonstrate that through the reprogramming of crRNA-tracrRNA pairings, SpCas9 can selectively repurpose diverse RNAs as crRNAs, thereby initiating CRISPR functions. With the information of these findings, they created an RNA sensor capable of co-opting endogenous RNA molecules as crRNAs. Notably, this approach successfully monitors the transcription levels of native genes in *Escherichia coli*, establishing a connection between the bacterial endogenous genetic network and an artificial gene circuit. This circuit functions as a programmable whole-cell biosensor. Furthermore, this strategy gives rise to a novel RNA sensing

technique, termed AGATHA, which proves effective in targeting the detection of SARS-CoV-2 RNA in vitro.

In contrast to well-known CRISPR RNA sensors like SHERLOCK, HOLMES, and DETECTR, which exhibit continuous cleavage of nonspecific single-stranded nucleic acids, Cas9 cleavage lacks signal amplification. Unlike Cas12a and Cas13, Cas9 can only cleave specific target DNA and remains bound to it, resulting in insufficient repetitive cleavage of the target DNA. To overcome these limitations in Cas9-directed RNA sensing, Y. Liu et al. introduced a novel in vitro transcription-based biosensing reporter, termed the Atypical gRNA-activated Transcription Halting Alarm (AGATHA) system.

The AGATHA system comprises a reporter DNA, fluorogenic DFHBI, purified Cas9, and reprogrammed tracrRNA, aiming to detect target RNA in an in vitro T7 expression system. Within AGATHA, the DNA expresses an inactive Broccoli RNA aptamer restrained by a 3'-end secondary structure (anti-Broccoli tail). Upon detecting the target RNA, activated Cas9 intervenes, halting the transcription of the 3'-end secondary structure. This interruption enables continuous transcription of a functional aptamer. The functional aptamer subsequently binds to DFHBI, producing an amplified fluorescent output signal. This innovative design aims to address the shortcomings of Cas9-based RNA sensing by employing a transcription-based strategy for signal amplification.

Bacterial detection

In the amplification-free detection reliant on the binding activity of dCas9 effectors, optical readout technologies like surface-enhanced Raman scattering (SERS) and fluorescence in situ hybridization (FISH) were employed. SERS refers to a phenomenon wherein Raman signals from molecules intensify in proximity to SERS-active surfaces [17–19]. Kim et al. established a SERS detection technique employing the CRISPR/dCas9 system to identify multidrug-resistant (MDR) bacteria, including *Staphylococcus aureus, Acinetobacter baumannii,* and *Klebsiella pneumoniae.* [20]. Mutations in chromosomal genes or the acquisition of mobile genetic elements such as plasmids and bacteriophages can cause multidrug resistance in bacteria [21]. Identifying antimicrobial resistance (AMR) genes in bacteria is crucial. PCR-based detection of multidrug-resistant (MDR)

bacteria through AMR gene sequences is common. However, PCR methods, while highly sensitive, entail lengthy operating times, complex primer design, extensive reagents, a demand for high technical expertise, and substantial equipment requirements.

In the identification of multidrug-resistant (MDR) bacteria like Staphylococcus aureus, Acinetobacter baumannii, and Klebsiella pneumoniae, dCas9/gRNA ribonucleoproteins (RNPs) were engineered to target these bacteria. These modified dCas9/gRNA complexes were then linked with Au-coated magnetic nanoparticles (Au MNPs). This hybridized Au MNP-dCas9/gRNA complex was exposed to MDR bacterial genes, capturing these superbugs. Strong SERS signals were observed from the captured multidrug-resistance genes on the Au MNP-dCas9/gRNA probes following Raman dye integration and magnetic separation.

Kim et al. effectively detected MDR bacteria from clinical specimens with femtomolar-level sensitivity, bypassing the need for gene amplification. Moreover, they diagnosed MDR bacteria in infected mice using this method. Additionally, a three-dimensional (3D) nanopillar array was utilized to trap MDR bacteria on-site, facilitating continuous detection through the CRISPR/dCas9-mediated SERS test. The authors predict that this novel technology could be developed to identify a wide range of diseases, aiding in early-stage patient treatment for bacterial diseases while reducing superbug multiplication and transmission [22].

Mutations Detection

Hajian et al [23]. developed the CRISPR–Chip, merging CRISPR–Cas9's gene targeting with a graphene-based field-effect transistor (gFET) for sensitive detection. This biosensor swiftly detects intact genomic DNA from specific transfected cells, using a catalytically deactivated Cas9 complex on modified graphene. The CRISPR–Chip's gFET alters electrical properties upon the selective pairing of target DNA with the complex, generating an electrical signal. It enables specific, reagent-free detection of DNA, all within a compact setup.

The first CRISPR–Chip was utilized for identifying genetic mutations associated with Duchenne muscular dystrophy (DMD). The CRISPR–Chip successfully identified the deletion of two specific target sequences in DMD patients without requiring any pre-amplification. These proof-

of-concept studies indicate that CRISPR–Chip has the potential to eliminate the necessity for sequence amplification in the analysis of hereditary diseases. This is because the genomic material needed for CRISPR–Chip analysis can be obtained using the readily available buccal swab method [23].

The CRISPR-Chip, demonstrated by the authors, showcased a lower Limit of Detection (LOD) compared to previously reported amplification-free technologies used for detecting target sequences across the genome. While an alternate method utilizing plasmon resonance imaging with increased sensitivity exists, it demands DNA sample fragmentation via sonication, multiple DNA probes, and bulky optical equipment for sequence-specific detection. In contrast, the CRISPR-Chip, as illustrated, offers numerous advantages over alternative techniques like gold nanoparticle microarrays and surface plasmon imaging for amplification-free genomic analysis.

The process for analyzing samples with the CRISPR-Chip involves a simple three-step procedure (calibration, incubation, and rinsing) and requires only a portable digital reader and reaction buffer. This straightforward process positions it favorably for practical use in point-of-care settings.

Swift and uncomplicated techniques for identifying specific genes at the single-nucleotide level have the potential to extend genetic research and diagnostic applications beyond conventional laboratory settings. In a recent study, Balderston et al. [24] introduced an innovative biosensor intended for the electronic detection of unamplified target genes. This pioneering approach involves liquid-gated graphene field-effect transistors that integrate an RNA-guided catalytically deactivated Cas9 bound to a graphene monolayer.

In the experiment employing unamplified genomic samples from patients, diverse electrical responses were measured. The biosensors demonstrated the capability to differentiate, within an hour, between wild-type and homozygous mutant alleles differing by a single nucleotide. Additionally, by utilizing a guide RNA–Cas9 orthologue complex targeting genes within the protospacer-adjacent motif (PAM), the biosensor effectively discerned between homozygous and heterozygous DNA samples from individuals with sickle cell disease. Most importantly the mentioned biosensor was also shown to be effective in rapidly screening guide RNA–Cas9

complexes, identifying those that optimize gene-targeting efficiency. These findings underscore the potential of the biosensor by Balderston et al. for swift and accurate genetic analysis in real-world scenarios[24].

CRISPR/Cas13-based Biosensors for Disease Diagnostics

Viral RNA detection

The SHINE method offers two types of readouts, namely lateral flow, and in-tube fluorescence, each with its own set of advantages and drawbacks related to equipment needs and sample batch size [25] . The in-tube fluorescence readout allows for parallel imaging of numerous samples, but it requires a blue light-emitting device. The adoption of portable transilluminators removes the necessity for bulky or costly fluorescent readers. Additionally, the in-tube fluorescence readout, coupled with a smartphone application, facilitates automated result interpretation, ensuring both speed and impartiality. SHINE is especially fitting for community surveillance testing due to its combination of straightforward preparation, user-friendly features, ample sensitivity, and quick turnaround time [26,27].

SHINE and SATORI allow for a single mismatch between guide and targeted sequences. This incompatibility tolerance could help expand their applicability beyond detecting RNA of viruses to identify novel and known point mutations [12].

Moreover, Fozouni et al. [28] also developed a cost-effective CRISPR/Cas13a-based method to directly detect SARS-CoV-2 RNA quantitatively without amplification in 30 minutes. Fluorescence could be easily measured in real-time instead of measuring the end-point using a smartphone camera in a small device with laser lighting and collecting lens, demonstrating the convenience and mobility of the diagnostic assay (Fig. S2). The high sensitivity of mobile phone cameras, along with their connection, GPS, and figures-processing capabilities have made them tempting instruments for point-of-care disease detection with minimal resources [29].

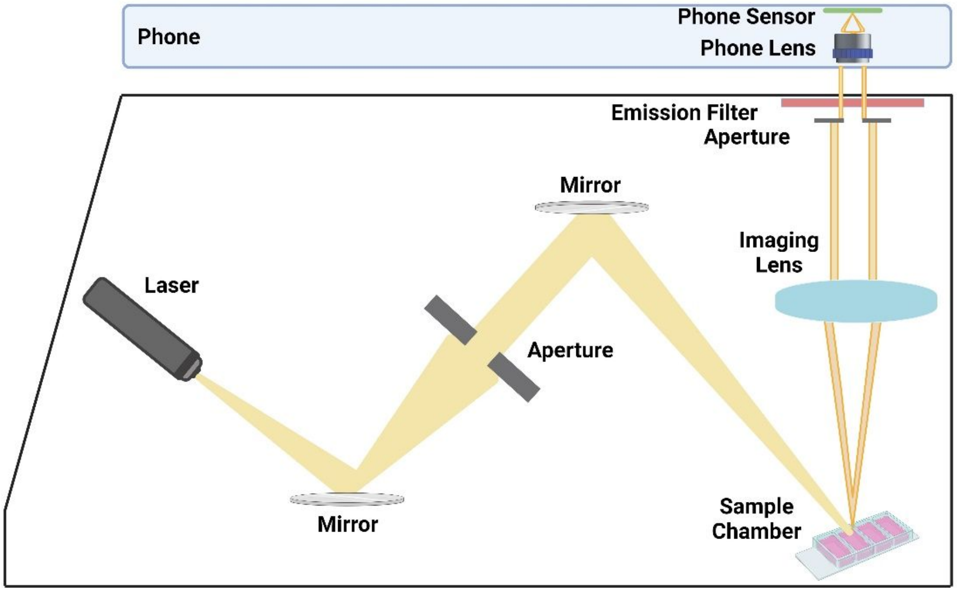

Figure S2. *The diagram outlines how a mobile phone-based microscope detects fluorescence in a Cas13a assay. An LED light source emits light through filters onto the sample. A lens magnifies the emitted fluorescent light, filtered for quality, and captured by a mobile phone camera held in place by an adapter. This setup enables accurate fluorescence detection and analysis via a mobile phone. Reproduced with permission from ref* [28]. *Copyright 2020 Elsevier Inc.*

Fluorescence data can be readily converted into virus loads immediately for quantification, distinguishing it from similar CRISPR assays like CRISPR-COVID, which employ isothermal amplification for high sensitivity but only qualitative information [30]. . By combining multiple crRNAs binding to distinct sections of the genome, detection of target viral RNA in the attomolar range becomes possible, preventing the loss of detection due to natural mutations in viral genomes. The crRNAs are tested against various respiratory-related RNA viruses, including coronaviruses causing respiratory illnesses in human hosts [31] and influenza viruses. None of the other viral RNA samples exhibit a detectable signal above the RNP complex background, demonstrating specificity. This method can offer a rapid, precise, portable, and affordable SARS-CoV-2 and potentially other viral RNA detection alternatives at the point-of-care by reprogramming the crRNA to induce Cas effector specificity. When combined with various Internet of Things technologies, including fast contact tracing and cloud-based platforms, this mobile diagnostic device might play a key role in public health. Another technology developed by Wang et al. [32] has recently emerged to address both issues related to amplification-free and point-of-care detection by using a single molecule digital CRISPR (dCRISPR)-based method in microfluidic chips to

provide sensitivity up to 2 aM of SARS-CoV-2. The Cas13a-crRNA complex is immobilized on femtoliter-sized microwells, limiting fluorescence spread to improve local signal intensity. This is particularly useful as a shorter detection time enables the implementation of the device in point-of-care. However, this technology falls short of addressing the benefits of integrating a multiplex detection assay.

Other strategies for point-of-care detection such as multiplex detection have also been implemented to develop a rapid electrochemical biosensor, named E-CRISPR, targeting two different regions of the SARS-CoV-2 RNA genome in a clinical sample [33]. This method involves exposing the Cas13a-crRNA-target RNA assembly on the immobilised nonspecific reporter RNA on a gold nanostructured electrode, leading to signal amplification. The improvement in sensitivity to the attomolar level is due to the combination of the intrinsic sensitivity of electrochemical methods and the collateral cleavage of the Cas enzyme. This can potentially be beneficial for the early detection of diseases when there is a low viral gene load, as well as reducing the possibility of obtaining false-negative results. Another electrochemical biosensor has been developed in a similar manner to target the ORF and S genes of SARS-CoV-2 in salivary samples by depositing a novel nanocomposite and gold nanoflower on an electrode [34]. The integration into an all-in-one type cartridge to enable the direct use of complex samples like saliva will boost its usefulness for on-site detection.

Apart from the detection of SARS-CoV-2 RNA, CRISPR/Cas13 has also been utilised for other RNA viruses like Ebola. Direct detection of unamplified Ebola RNA samples was achieved by integrating a Cas13a-mediated DNA roller (Cas-Roller) to amplify and generate fluorescence signals with excellent sensitivity whilst providing minimal interference in human blood and serum samples [35]. There are two processes in Cas-Roller, which are CRISPR-Cas13a and DNA roller reactions, are effectively linked using a hairpin DNA-Au nanoparticle structure labeled with rArU. When the crRNA-Cas13a complex identifies and attaches to the target RNA, it triggers Cas13a activation, resulting in the cleavage of neighboring rArU. This cleavage generates single-stranded (ss) DNA-Au nanoparticles, initiating the subsequent DNA roller reaction and producing an amplified fluorescent signal. Remarkably, the Cas-Roller assay circumvents the necessity for reverse transcription of viral RNA to DNA and intricate nucleic acid pre-amplification. This approach addresses concerns regarding cross-contamination and potential amplification bias.

Furthermore, given the independence of the subsequent Roller reaction from the target sequence, this method can be adapted to detect any RNA by modifying the crRNA hybridization regions. Consequently, it showcases considerable potential as a rapid, precise, and straightforward approach for various RNA assays, including the detection of other infectious agents. Moreover, the outcomes may be examined using the RGB values, red, green, and blue, that the iPhone operating software (iOS), Palette Cam, reads. Simple visualization and analysis of the RGB values allow for easy implementation for rapid detection. Furthermore, modification of the crRNA can broaden the scope of this technique to identify different RNA viruses in a rapid, accurate, and simple manner.

Cancer-associated RNA detection

MicroRNAs (miRNAs) are matured noncoding RNA that bind to specific messenger RNA sites, inducing degradation to regulate gene expression. Dysregulation of miRNA has been associated with certain cancers, making it a potential tumour biomarker [36]. Identifying miRNAs is challenging due to small size, low abundance, and high homology. Bruch et al. [37] developed a CRISPR/Cas13a-driven signal amplification on a microfluidic electrochemical biosensor capable of detecting picomolar levels of the putative brain tumour marker miR-19b in blood samples from brain cancer patients (Fig. S3). The test was optimised through variations in incubation duration and concentration. It was discovered that shaking the solution inhibited the enzyme’s catalytic activity and alleviated reagents diffusion limitations by promoting the removal of crRNA. Furthermore, the Cas13a-crRNA complex could not sufficiently hybridise with mismatched miRNAs that differ from miR-19b by single or multiple nucleotides, preventing the stimulation of the enzyme’s catalytic activity due to its high specificity. This biosensor chip was assessed by evaluating blood samples from young patients with medulloblastoma at various stages. By studying the same samples using a typical quantitative real-time polymerase chain reaction (qRT-PCR) approach, the results of this biosensing chip were confirmed to be in good agreement, suggesting that the biosensor chip may be employed to detect miRNA-related illnesses.

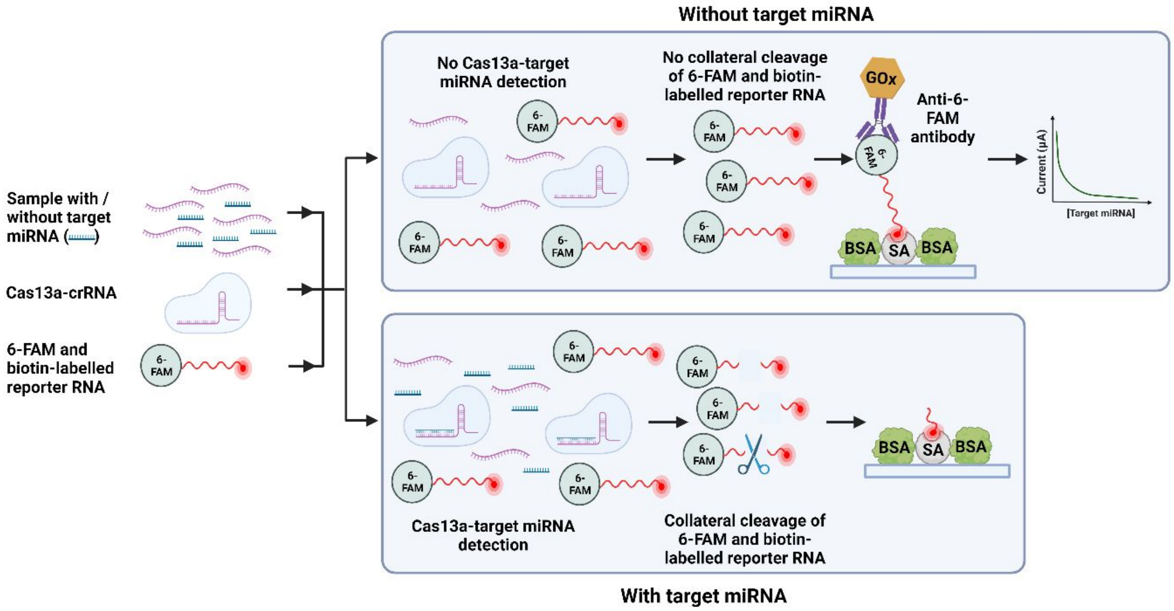

***Figure S3**. Schematic diagram for the diagnosis of miRNA using the CRISPR/Cas13 System and an electrochemical microfluidic platform. The activation of the Cas13a enzyme involves combining target-specific crRNA, biotin, 6-FAM (6-fluorescein amidite)-tagged reporter RNA, and the sample of interest. Cas13a is subsequently triggered upon encountering target miRNAs, inducing collateral cleavage of adjacent reporter RNA.. Created with BioRender.com with permission from ref* [37]*. Copyright 2019 WILEY-VCH Verlag GmbH & Co.*

Similarly, the work was further improved by replacing the probe with a tetrahedral DNA framework in a CRISPR/Cas13a-based electrochemical biosensor (CRISPR-E) [38]. Using the self-assembled three-dimensional DNA nanostructure, the tetrahedral DNA framework avoids entanglement and lodging in one-dimensional linear strands and two-dimensional hairpin structures that can hinder the cleavage of CRISPR/Cas systems, further lowering the sensitivity in Bruch et al. [37]. Ultrasensitive detection of unamplified miRNA-19b as low as 10 pM can be achieved selectively using this improved and simplified CRISPR-E platform on a chip, highlighting its potential role in advancing point-of-care diagnostics.

Cancer molecular markers detected in traditional tissue biopsy enable the detection of original tumours or the grading of metastatic lesions. Liu et al. [39] created a plasmonically-enhanced fluoroimmunoassay (p-FLISA) with CRISPR/Cas13 for the qualitative and quantitative measurement of RNA in cells (Fig. S4). To improve sensitivity, they employed plasmonic-fluor

as a nano reporter giving about 3700-fold greater fluorescence emission in place of the standard fluorochrome or enzyme used in the traditional fluorophore-linked immunosorbent assay (FLISA). The use of p-FLISA was examined in tissue samples for long noncoding RNA (lncRNA) H19 detection using a human ovarian cancer xenograft mouse model from immunodeficient athymic nude mice to develop human cancer cell lines spontaneously in vivo with the same behaviour. This results in a tumour microenvironment with many oncogenic genes, including lncRNA H19. The standard curve was used to calculate the number of lncRNA H19 in each isolated RNA sample. The number of lncRNA H19 detected by qRT-PCR in the same tissue sample exhibited an excellent correlation with samples detected by p-FLISA.

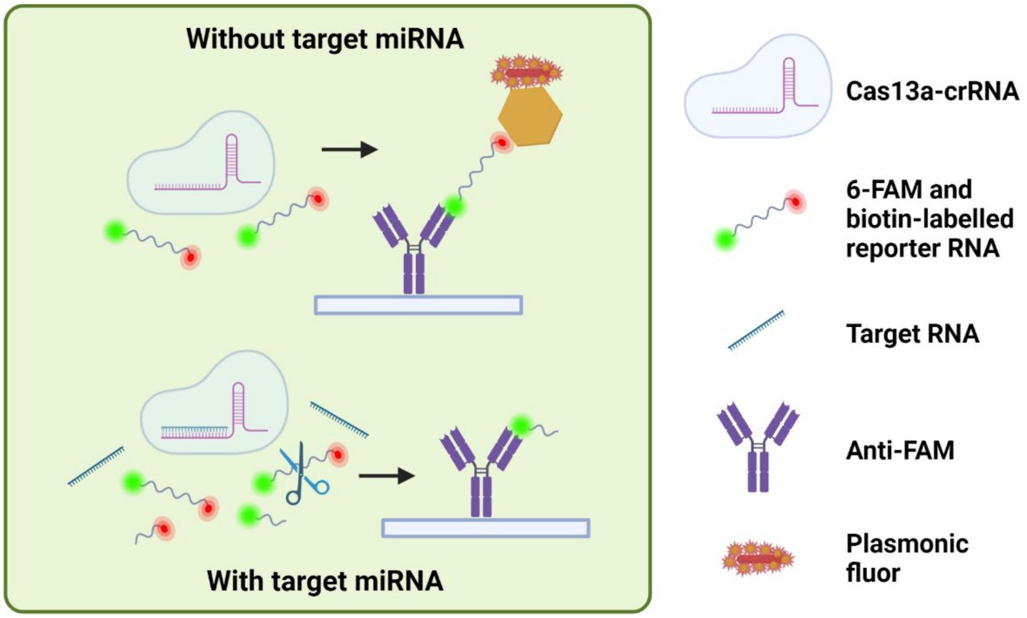

Figure S4. *Illustration of CRISPR/Cas system biosensing technology combined with plasmonically enhanced amplification-free detection of target RNA. Created with BioRender.com with permission from ref* [39] *. Copyright 2021 WILEY-VCH Verlag GmbH & Co.*

CRISPR/Cas12-based Biosensors for Disease Diagnostics

Cancer-associated cell-free DNA (cfDNA) detection

Cancer cells release cfDNA into the blood plasma during apoptosis and necrosis, reaching significant concentrations that can indicate early- or late-stage cancer [40]. Analysing cfDNA as a

non-invasive cancer biomarker holds promise for diagnostics, therapeutics and prognosis in cancer patients. Despite its potential, cfDNA is present in low concentrations in physiological fluids, necessitating an ultrasensitive approach for detection before widespread clinical use.

In 2021, an amplification-free CRISPR/Cas12a-based metal-enhanced fluorescence (MEF) biosensor with DNA-functionalised AuNP, utilising dual detection systems, was developed for cfDNA detection [41]. This system effectively replaced target amplification with a signal enhancer based on DNA-functionalized AuNP-based MEF, providing outstanding fluorescence intensity and enabling visual inspection of colour changes due to the activation of CRISPR/Cas12a in the presence of target cfDNA (Fig. S5). Due to the plasmonic effects of AuNPs, the colour of MEF shifted from purple to red-purple with the degradation of ssDNA between AuNP and fluorophore. The nanosensor exhibited high sensitivity, detecting the BRCA-1 gene, a biomarker associated with breast cancer, down to femtomolar levels, and achieved rapid detection time in less than 30 minutes. This nanosensor shows promise as a point-of-care alternative for cancer detection. However, further investigations are crucial to assess its utility in detecting other cfDNAs, genomic DNA, and viral DNA.

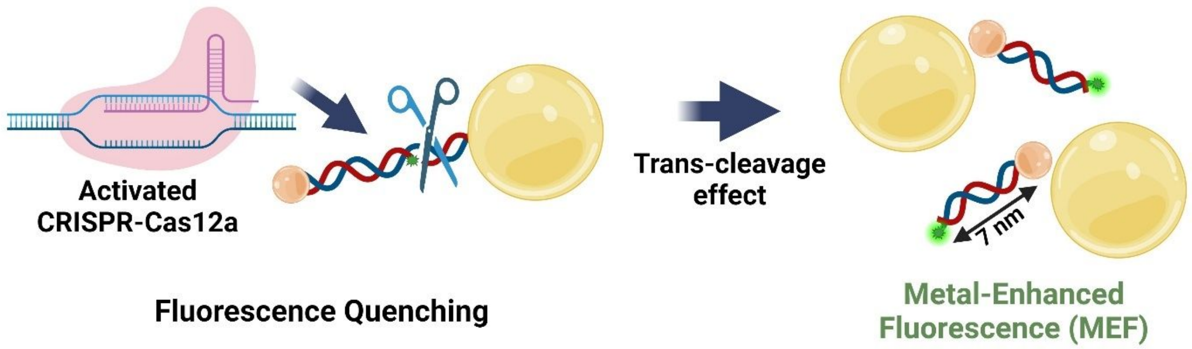

Figure S5. Detection of cfDNA using metal-enhanced fluorescence (MEF) with DNA-functionalized Au nanoparticle. AuNP pairs of distinct sizes (20 and 60 nm) were linked by a 7 nm long dsDNA and a 2 nm long ssDNA to induce MEF and fluorescence quenching, respectively, with and without target DNA. Fluorescein isothiocyanate (FITC) was linked to the end of the dsDNA not bound to the 60 nm-sized AuNPs, remaining in a quenched state due to its proximity to the 60-AuNPs. Upon the presence of target cfDNA, the activated CRISPR/Cas12a complex cleaves the ssDNA between 20- and 60-AuNPs, causing dissociation of the 60-AuNPs from FITC, as well as the 7 nm DNA-functionalized 20-AuNPs. This generates a fluorescence signal directly proportional to the concentration of target cfDNA, as low as in femtomolar ranges. Created with BioRender.com with permission from ref [41 42]. Copyright 2020 American Chemical Society.

Multiple Targets Detection

The qualitative and quantitative identification of biomarkers is crucial for diagnosis. However, since a single biomarker might be symptomatic of more than one illness, detecting a single biomarker is inadequate to identify a disease [43–45]. Utilizing multiplexed detection of biomarkers, which involves simultaneously identifying numerous biomarkers in a single test, enhances diagnostic accuracy by providing scientifically precise information. This approach also improves diagnostic efficiency through faster processing, minimizing the likelihood of errors. [46,47]. In many diagnostic instances, comprehensive and simultaneous testing for multiple targets, known as multiplexing, is desired due to its ability to provide more information at a greater throughput and a lower cost per target.[48,49]. Endogenous biomarkers, whether proteins or nucleic acids, might face limitations in body circulation concerning their quantity or stability. This prompts the development of engineered 'synthetic biomarkers' aimed at boosting analyte production, thereby improving signal-to-noise ratios by tapping into the chemical composition of the diseased environment. Achieving precise categorization of various dynamic disease states in clinical settings relies on highly multiplexed readouts in molecular diagnostics [50–52].

CRISPR multiplex detection refers to the use of the CRISPR-Cas system for detecting multiple target sequences simultaneously. Dahlman et al. and a team of researchers utilized chemically solidified DNA for molecular barcoding. They employed CRISPR-Cas nucleases to achieve sequence-specific readouts, enabling the multiplexing of synthetic biomarkers for precision diagnostics at point-of-care (POC). [53]. They engineered a collection of high-throughput, programmable in vivo nanosensors intended for non-invasive illness detection and urine analysis by integrating advanced technologies. This unified synthetic biomarker technology enables multiplexed in vivo sensing of proteolytic activity in the microenvironment. The sensors are encoded using synthetic DNA barcode intermediates and utilize CRISPR-based amplification for paper readout, showcasing a comprehensive approach to advanced diagnostic capabilities.

CRISPR with Sequencing

Despite the increasing utilization of next-generation sequencing (NGS) technology, targeted sequencing in specific genomic regions remains essential for identifying a broad spectrum of hereditary diseases. Target enrichment proves effective in minimizing the cost and time of

sequencing. Recently, several CRISPR-based nucleic acid amplification-free sequencing methods for target enrichment, coupled with various NGS platforms, have emerged. This serves as an adjunctive tool to enhance NGS analytical capabilities, enabling indirect detection of nucleic acids. [54]. Short tandem repeats (STRs), often called microsatellites, constitute approximately 3% of the genome, featuring repeating motifs spanning 2 to 12 base pairs in length. To date, over 50 genetic diseases have been associated with expansions in these STR regions [55]. The pivotal role of STR in disease pathology is evident, yet their investigation poses considerable methodological challenges. Among the molecular diagnostic methods used for studying repeat expansion disorders, two prominent approaches involve genotyping STRs through Southern blot analysis and employing triplet repeat primed PCR [56–58]. The clinical application of the Southern blot technique, often deemed the gold standard for identifying substantial repeat expansions, encounters several challenges. These include high costs, the requirement for considerable quantities of high-quality DNA, limited sensitivity, lengthy processing time, and inherent procedural complexity [59]. Repeat-primed PCR offers both cost-effectiveness and enhanced efficiency compared to other methods. However, a major challenge it faces involves a significant increase in PCR stutter attributed to the introduction of artificial deletions and insertions within highly repeated regions during the amplification process [60.] Moreover, the likelihood of successful PCR amplification significantly diminishes in regions characterized by high GC content. Furthermore, PCR amplification in repetitive regions can induce allele bias, potentially resulting in misinterpretations of results [61].

CRISPR-Cas9 technology enables the genotyping of STR expansions and the detection of somatic mosaicism and repeat motif interruptions. This capability highlights the limitations of conventional genotyping methods in capturing the dynamic nature of STRs in their extended state, as these methods provide only size estimates rather than detailed information about repeat architecture.[62]. Overall, STR sequencing using CRISP-Cas9 produced more informative STR-single-nucleotide polymorphism haplotypes than prior STR studies. The amplification-free strategy utilizing the CRISPR-Cas9 system offers a comprehensive assessment of nucleotide content, repeat expansion sizes, and potential modifiers in emerging diseases associated with repeat expansions. Through CRISPR-Cas9 technology, it becomes feasible to genotype STR expansions and identify somatic mosaicism and interruptions within repeat motifs. This capability exposes the limitations of

conventional genotyping methods, which merely offer size estimations without detailed information about the dynamic nature and architecture of extended STRs [63–65].

Non-Nucleic Acid (NNA) Target Detection

Aptamers are short 20-60 nucleotide sequences that are specific and bind to NNA, mediating the release of a blocker. This blocker is an activator DNA complementary to gRNA for Cas enzymes. The aptamer binding affinity, evaluated by dissociation constant, was found to be only in the picomolar to micromolar range. This suggests that the aptamer must be paired with other approaches to achieve excellent sensitivity [66]. When combined with CRISPR/Cas technologies, aptamers can be exploited for ultrasensitive and highly specific disease biomarker detection [67]. More recently, CRISPR/Cas14a1 aptamer-based amplification-free platforms (ACasB) have been developed for live detection of *Staphylococcus aureus* with high sensitivity and specificity [68]. Altering the sequence of aptamers allows for the detection of other pathogenic microorganisms, not only in disease diagnostics but also in environmental monitoring and food safety. Future work should focus on reducing the detection limit to levels comparable to other CRISPR/Cas methods and simplifying the detection method.

Employing antibodies for NNA target recognition in CRISPR/Cas-based biosensors has also shown enormous possibilities in sensing low levels of NNA targets with high sensitivity. In a study published by Duan and colleagues [69], CRISPR/Cas12a-mediated DNA extraction and amplification-free, highly direct and rapid biosensor (CATCHER) was developed using antibodies, DNase I, and colloidal gold nanoparticles for the detection of *Salmonella typhimurium,* which causes gastrointestinal diseases in humans, within 2 hours. The universality of this platform allows for detecting other NNA targets like viruses by simply changing the antibodies. The practicability of this biosensor becomes important for the rapid, on-site detection of infectious targets at low concentrations. Nevertheless, antibody-based sensors used in rapid on-site detection have some limitations due to their poor chemical stability [70]. Some modifications may also be needed to compress the workflow into a one-pot reaction for further simplification and ease of use.

aTFs are naturally occurring effector molecules derived from bacteria that have both a DNA binding domain and an effector sensory domain [71–73]. This system mainly relies on the change in

allosteric binding activity between isolated aTFs and their aTF binding site (TFBS) in response to the presence of its NNA targets. Stable biosensors with high sensitivity have been successfully constructed for uric acid and tetracycline by employing isolated aTFs as recognition elements that can regulate gene expression *in vitro* [74–76]. Unfortunately, the recognition components employed in those studies are difficult to acquire for common NNA targets. Therefore, it is essential to note that more computational studies on bacteria and protein engineering approaches need to be further utilised to ensure the full potential of aTFs in detecting NNA molecules is explored.

Recent advancements in CRISPR and CRISPR/Cas systems have facilitated the development of highly sensitive and efficient biosensing platforms for the detection of small NNA molecules using diverse aTFs. One study introduced by Mahas et al. developed a rapid and sensitive platform for detecting small molecules using an aTF-controlled expression of a CRISPR array combined with Cas12a activity. The above system enables a rapid, sensitive, and specific detection of tetracycline antibiotics through a robust fluorescent signal triggered by the presence of the target molecule. The platform exhibits high accuracy in detecting various tetracycline antibiotics and is complemented by simplified readouts, a handheld visualizer, and a mobile app for facile data interpretation. Moreover, the platform's amenability to lyophilization ensures convenient storage and distribution, making it well-suited for field applications [77].

Liang et al. [78] had also combined an aTF-based small-molecule sensing platform with the CRISPR/Cas12-mediated nucleic acid detection system, named CaT-SMelor (CRISPR-Cas12a and aTF mediated small molecule detector), in another study. They demonstrated the platform's ability to rapidly and sensitively detect various small molecules related to metabolic diseases, antibiotic residues, and food preservatives. Moreover, the system proved efficient and highly accurate in swiftly analyzing uric acid levels in clinical human serum samples, crucial in diagnosing conditions like gout. These findings suggest that the developed methodology holds significant promise for detecting and quantifying diverse small molecules across various applications.

In a study led by Wang et al[79] ., a nano-biosensor utilizingthe cleavage activity of CRISPR-Cas12a was designed for the detection of non-nucleic acid targets. The research utilised two approaches

to integrate biosignals with nanomaterials, resulting in a versatile nano-biosensor with CRISPR-Cas12a cleavage activity. This sensor demonstrated quantitative detection capabilities for diverse non-nucleic acid targets. The study employed a highly sensitive fluorescence sensor (UCNPs-Cas12a) and a colourimetric sensor (hydrogel-MOF-Cas12a) to provide intuitive results. The detection system used upconversion fluorescence, converting non-nucleic acid targets (estradiol and prostate-specific antigen) into a DNA signal through aptamer recognition and amplifying it via catalyzed hairpin assembly (CHA) to generate dsDNA (dsDNACHA) that activated CRISPR-Cas12a. The inclusion of upconversion alternative fluorophores and CHA further amplified the signal, enhancing detection sensitivity. On the other hand, the CRISPR-Cas12a colourimetric sensor is constructed with a DNA hydrogel-encapsulated MOF biomimetic enzyme, induced controlled lysis of the DNA hydrogel-coated MOF when combined with nanomaterials, releasing MOF-catalysed hydrogen peroxide for colour development. Although slightly less sensitive than the fluorescence method, this colourimetric approach allows for direct observation, making it applicable across various fields.

***Table S2**. Summary of different strategies of amplification-free CRISPR/Cas system for various analytes.*

Method	Cas Type	Targets	Signal read-out	LOD	Sample to answer time (min)	Ref
CRISPR enhanced E-DNA sensor	Cas9	PB-19	Electrochemical	100 fM	N.S	[20]
Reprogrammed of crRNA-tracrRNA	Cas9	SARS-CoV-2 RNA	FL	N.S	10	28

hybridization						
(dCas9)-mediated SERS assay	Cas9	MDR (*S. aureus, A. baumannii, K. pneumoniae*)	SERS	14.1 fM<br>9.7 fM<br>8.1 fM	30	[22]
CRISPR–Chip gFET	Cas9	*bfp* gene	Electrical	1.7 fM	15	[23]
CRISPR-based gFET	Cas9	SNP	Electrical	10–60 ng μl^{-1}	40	[24]
CRISPR-Cas Sequencing	Cas9	STR	Sequencing	N.S	N.S	[62]
SATORI	Cas13	SARS-CoV-2	FL	~10 fM	< 5	[45]
SHINE	Cas13	SARS-CoV-2	FL	10^5 cp/μL	50	[53]
CRISPR-Cas13a and	Cas13	SARS-CoV-2	FL	~100 copies/μL	< 30min	[28]

mobile phone microscopy						
E-CRISPR	Cas13	S and Orf1ab gene	FL	2.5 ag/µL 4.5 ag/µL	180	[64]
dCRISPR	Cas13	SARS-CoV-2	FL	2 aM	50	[58]
Cas-Roller	Cas13	Ebola virus RNA	FL	291 aM	~40	[61]
CRISPR/Cas13a-driven microfluidic electrochemical	Cas13	miR-19b	Electrochemical	10 pm	<240	[63]
CRISPR-E	Cas13	miRNA-19b	Electrochemical	10 pM	60	[64]
p-FLISA	Cas13	IL-6	FL	~11.2pgml –	20	[39]
CRISPR-SERS	Cas12	HBV	SERS	1 fM	30 - 40	[77]

CRISPR/Cas12a droplet assay	Cas12	Viral RNA	FL	100 fM	60	42
CRISPR/Cas12a-based metal-enhanced fluorescence (MEF)	Cas12	cfDNA	FL		< 30	78
CRISPR/Cas 12a Aptasensor	Cas12	Ampicillin	FL	0.01nM	30	66
CRISPR/Cas 12a Aptasensor	Cas12	CD63	FL	3×10^3 particles/μL	15	115
CATCHER	Cas12	*Salmonella typhimurium*	FL	7.9×10^1 CFU/mL	⩽ 120	111
aTF-controlled expression of	Cas12	Tetracycline antibiotics	FL	2 μM	120	118

CRISPR/Caas12a array						
CaT-SMelor	Cas12	Uric acid and *p*-HBA	FL	25 nM<br>1.8 nM	40	78
UCNPs-Cas12a/hydrogel-MOF-Cas12a	Csa12	E2 and PSA	Fluorescence/colorimetric	0.015 ng/mL<br>0.14 ng/mL	40	123
Lab-On-a-Chip	Cas12	SARS-CoV-2	Electrochemical	2.3 viral RNA copies/μL	60	80
CRISPR/Cas14a1 aptasensor	Cas14	*Staphylococcus aureus*	FL	400 CFU/ml	20	110

Detection Read-out Methods

Different readout methods have been developed for amplification-free CRISPR/Cas-based diagnostics in point-of-care. Fluorescence and lateral flow assay are the predominant techniques employed for CRISPR/Cas diagnostic platforms.

Most CRISPR/Cas-based diagnostic approaches for fluorescence-based assays use single-stranded FRET probes containing a fluorophore and a quencher. Upon target recognition, Cas12 or Cas13 unleashes trans-cleavage activity, separating the fluorophore from close proximity to the quencher,

inducing an increase in fluorescence intensity. Fluorescence-sensing platforms have various advantages, including an enhanced signal-to-noise ratio compared to other optical techniques. However, they rely on bulky and expensive instruments that are often inappropriate and less robust for resource-limited settings [74]. A handheld fluorescence reader is, therefore, an appealing choice for bringing fluorescence-based diagnostics into the field to achieve greater precision, compactness, and high energy efficiency. It minimises the use of expensive optical components that are usually more specialized and less amenable to adoption at the point-of-care [81].

In the early phases of CRISPR/Cas-powered biosensors, colourimetry was used as a readout for assessing nucleic acid recognition. With constant improvements in research methodologies, the difference in performances, especially sensitivity, has substantially increased, showing promise in application diagnostics [82]. Nonetheless, colourimetric sensors encounter the same issues of false positives and negatives generated by the instability of the reporter molecule and perhaps a more subjective signal response [83]. Lateral flow assays are an excellent alternative to overcome the limitations presented by fluorescence-based detections due to properties such as ease-of-use, visual detection, cost efficiency, stability, and simplicity [84]. They are colourimetric sensors that detect changes in colour caused by the presence of analytes. On the other hand, these traditional paper-based platforms are inefficient, which could result from factors such as slow diffusion rates, irregular or unpredictable flow paths, or inadequate lateral transport mechanisms. This can impact their accuracy, speed, or reliability in detecting or analyzing substances, rendering them unsuitable for the higher quantitative analyses necessary in clinical applications [85], such as in medical diagnostics, environmental testing, or food safety [86]. Particularly, it is difficult to quantify the readout accurately when the target concentrations are near the LOD of the assay. Nonetheless, the potential for the miniaturisation of these assays can recover the sensitivity lost through the lateral flow assay format for portable and rapid in-field analysis of small sample volumes.

Employing microfluidic methods, miniaturised devices integrating sample preparation, sensing, and reporting in single, easy-to-use units are becoming more feasible for low target concentrations in samples [142]The coupling of such devices with digital analysis and data-sharing platforms offers promise for patient-centric diagnostics and remote data access. Other less common signal detection methods, like electrochemical biosensors, have been reported.

For instance, Dai et al. [87] exploited the trans-cleavage activity of Cas12 and Cas13 to obtain a high signal without amplification via an electrochemistry-based platform with a low-cost transduction element and a disposable sensor. Additionally, alternate optical approaches based on SERS and SPR enable label-free detection of low nucleic acid levels utilising nanoparticles that can distinguish between single base-pair mismatches. The reliance on spectroscopic instrumentation, however, will most likely confine such techniques to clinical laboratories rather than point-of-care applications [88]. Naked-eye detection methods, including colourimetric and fluorescence under blue light, and mobile phone measurements [28,89,90], represent the current trends in signal detection methods where simple and automated detection has been favoured due to their cost-efficiency for bedside testing.

Strategies to improve the amplification signal of CRISPR/Cas system

An effective integrated biosensor must demonstrate competence in both target recognition and signal transduction for readout. The CRISPR/Cas system's strong ability to cleave target sequences provides heightened sensitivity, target specificity in identification, and signal amplification for biosensing applications. Typically, CRISPR/Cas biosensors employ a variety of signal transduction methods, including optical and electrochemical signals. In comparison to alternative techniques, fluorescence presents significant benefits in both qualitative and quantitative measurements. Nevertheless, a major hurdle faced by researchers in amplification-free CRISPR/Cas biosensors involves determining methods to enhance the readout signal of the CRISPR system without resorting to nucleic acid target amplification. This section explores the existing challenges and strategies employed to elevate the signal sensitivity of CRISPR/Cas systems in the context of the assay.

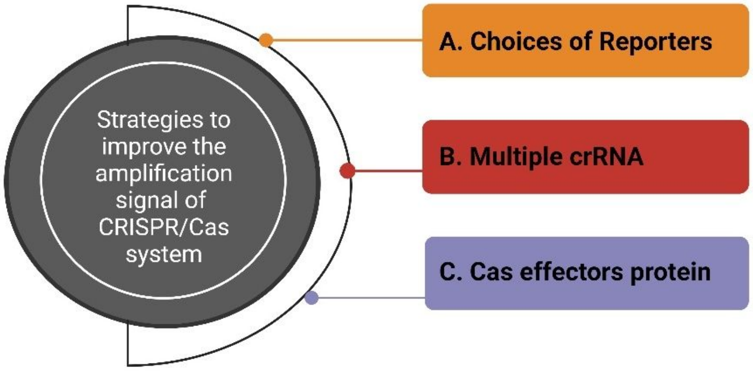

Figure S6. *Strategies to enhance signal strength in the CRISPR/Cas system.*

1. Choices of Reporters

Extensive CRISPR/Cas-based bioassays with superior analytical performance have been developed to date [91–93]. However, most fluorescence reporters are constrained by ssDNA-templated substrates used in previous studies. Although ssDNA reporters have been successful in diagnostic applications, the limited choices of short ssDNA may restrict their application and probe variety in diagnostics. By increasing the DNA substrate library for probes of Cas effector protein activity is critical. Unfortunately, only the fluorescence signals of a few reporters were closely analysed.

In a study by Liu et al., the cleavage characteristics of Cas12a were investigated across various fluorescent substrates. The design of single-stranded DNA (ssDNA) reporters was based on the distance between fluorophores and quenchers, determined by the number of bases. Additionally, double-stranded DNA (dsDNA) reporters were created using spacers within the secondary structure after annealing, while molecular beacon (MB) reporters were constructed based on the number of bases on the stem and loop. Comparative analysis under identical conditions was conducted, assessing several signal readout features among these fluorescent reporters, including real-time fluorescence curves, background noise, signal-to-background ratio, fluorescence recovery rate, and cleavage rate. The study concluded that, in identical conditions, MB reporters exhibit superior analytical performance compared to ssDNA and dsDNA reporters [94].

2. Multiple crRNA

CRISPR/Cas is a two-component complex system that uses the gRNA sequence to guide the Cas endonuclease to the target site [95]. Modifying the gRNA independently of the Cas protein provides convenience and flexibility in improving the CRISPR/Cas system as a biosensor. gRNAs have been designed to increase the overall stability, specificity, safety, and adaptability of the CRISPR system. Modifications aim to improve their stability and resistance to nuclease degradation, thereby increasing their efficiency. Furthermore, by minimising off-target effects, guide specificity has been increased. Doudna et al. and [96]. Published a two-crRNA-assisted amplification-free

CRISPR/Cas approach for viral detection. When the concentration of target RNA is low, targeting distinct sections of RNA by several RNPs is advantageous.. The combination of two crRNAs targeting two parts of the target RNA in the same process improves sensitivity. Consequently, the signal of the CRISPR/Cas system is enhanced when more target is detected [97].

3. Cas effectors protein

The turnover number (k_{cat}) directly relates to the maximum signal amplification rate achievable by CRISPR-Cas systems [98]. It indicates the effectiveness of the ternary complex comprising the target, crRNA, and Cas. The trans-cleavage activity of Cas proteins is highly sensitive to various factors, including the type of Cas protein employed, crRNA sequence, target site selection, target type, reporter type, reaction temperature, cofactor presence, inhibitor presence, additives, and even experimental procedures. Consequently, rigorous experimental standardization is crucial when comparing trans-cleavage activities across different Cas proteins or experimental setups [99–102].

For example, different studies have measured the kinetics of Cas12a homologs, reporting k_{cat} values ranging from 0.02 to 17 s^{-1}. AapCas12b, a thermophilic Cas12 from *Alicyclobacillus acidophilus*, exhibits a turnover number of 0.05–0.16 s^{-1} and has been employed in the one-pot SHERLOCK assay for SARS-CoV-2 detection. Another homolog, AsCas12a, has a turnover number of 0.5–1.3 s^{-1}. Due to differences in reaction conditions among these studies, determining the homolog with the highest activity is challenging. The trans-cleavage kinetics of Cas13 differ across its subtypes and homologs. In a study by East-Seletsky et al., the apparent cleavage rates of five Cas13a homologs (Lbu, Lwa, Ppr, Lba, and Hhe) were compared using a consistent set of spacers. Their findings highlighted LbuCas13a's superior trans-cleavage activity, detecting femtomolar concentrations of ssRNA activator, followed by LwaCas13a, which detected picomolar concentrations. Typically, the turnover number of Cas13 exceeds 1 s–1 [103].

Additionally, another approach to significantly reduce reaction volumes and enhance kinetics involves utilizing digital platforms. Implementing droplets or chip chambers with minimal volumes accelerates kinetics and amplifies sensitivity, as trans-cleavage activity markedly improves in micro-scale cleavage reactions.

References

(1) Hille, F.; Charpentier, E. CRISPR-Cas: Biology, Mechanisms and Relevance. *Philosophical Transactions of the Royal Society B: Biological Sciences* **2016**, *371* (1707), 20150496. https://doi.org/10.1098/rstb.2015.0496.

(2) Mojica, F. J. M.; Díez-Villaseñor, C.; García-Martínez, J.; Soria, E. Intervening Sequences of Regularly Spaced Prokaryotic Repeats Derive from Foreign Genetic Elements. *J Mol Evol* **2005**, *60* (2), 174–182. https://doi.org/10.1007/s00239-004-0046-3.

(3) Pickar-Oliver, A.; Gersbach, C. A. The next Generation of CRISPR–Cas Technologies and Applications. *Nat Rev Mol Cell Biol* **2019**, *20* (8), 490–507. https://doi.org/10.1038/s41580-019-0131-5.

(4) Nidhi, S.; Anand, U.; Oleksak, P.; Tripathi, P.; Lal, J. A.; Thomas, G.; Kuca, K.; Tripathi, V. Novel CRISPR–Cas Systems: An Updated Review of the Current Achievements, Applications, and Future Research Perspectives. *Int J Mol Sci* **2021**, *22* (7), 3327. https://doi.org/10.3390/ijms22073327.

(5) Makarova, K. S.; Wolf, Y. I.; Iranzo, J.; Shmakov, S. A.; Alkhnbashi, O. S.; Brouns, S. J. J.; Charpentier, E.; Cheng, D.; Haft, D. H.; Horvath, P.; Moineau, S.; Mojica, F. J. M.; Scott, D.; Shah, S. A.; Siksnys, V.; Terns, M. P.; Venclovas, Č.; White, M. F.; Yakunin, A. F.; Yan, W.; Zhang, F.; Garrett, R. A.; Backofen, R.; van der Oost, J.; Barrangou, R.; Koonin, E. v. Evolutionary Classification of CRISPR–Cas Systems: A Burst of Class 2 and Derived Variants. *Nat Rev Microbiol* **2020**, *18* (2), 67–83. https://doi.org/10.1038/s41579-019-0299-x.

(6) Koonin, E. v.; Makarova, K. S. Origins and Evolution of CRISPR-Cas Systems. *Philosophical Transactions of the Royal Society B: Biological Sciences* **2019**, *374* (1772), 20180087. https://doi.org/10.1098/rstb.2018.0087.

(7) Zhang, Y.; Qian, L.; Wei, W.; Wang, Y.; Wang, B.; Lin, P.; Liu, W.; Xu, L.; Li, X.; Liu, D.; Cheng, S.; Li, J.; Ye, Y.; Li, H.; Zhang, X.; Dong, Y.; Zhao, X.; Liu, C.; Zhang, H. M.; Ouyang, Q.; Lou, C. Paired Design of DCas9 as a Systematic Platform for the Detection of Featured Nucleic Acid Sequences in Pathogenic Strains. *ACS Synth Biol* **2017**, *6* (2), 211–216. https://doi.org/10.1021/acssynbio.6b00215.

(8) Wang, Q.; Zhang, B.; Xu, X.; Long, F.; Wang, J. CRISPR-Typing PCR (CtPCR), a New Cas9-Based DNA Detection Method. *Sci Rep* **2018**, *8* (1), 14126. https://doi.org/10.1038/s41598-018-32329-x.

(9) Zhang, B.; Xia, Q.; Wang, Q.; Xia, X.; Wang, J. Detecting and Typing Target DNA with a Novel CRISPR-Typing PCR (CtPCR) Technique. *Anal Biochem* **2018**, *561–562*, 37–46. https://doi.org/10.1016/j.ab.2018.09.012.

(10) Zhang, B.; Wang, Q.; Xu, X.; Xia, Q.; Long, F.; Li, W.; Shui, Y.; Xia, X.; Wang, J. Detection of Target DNA with a Novel Cas9/SgRNAs-Associated Reverse PCR (CARP) Technique. *Anal Bioanal Chem* **2018**, *410* (12), 2889–2900. https://doi.org/10.1007/s00216-018-0873-5.

(11) Quan, J.; Langelier, C.; Kuchta, A.; Batson, J.; Teyssier, N.; Lyden, A.; Caldera, S.; McGeever, A.; Dimitrov, B.; King, R.; Wilheim, J.; Murphy, M.; Ares, L. P.; Travisano, K. A.; Sit, R.; Amato, R.; Mumbengegwi, D. R.; Smith, J. L.; Bennett, A.; Gosling, R.; Mourani, P. M.; Calfee, C. S.; Neff, N. F.; Chow, E. D.; Kim, P. S.; Greenhouse, B.; DeRisi, J. L.; Crawford, E. D. FLASH: A next-Generation CRISPR Diagnostic for Multiplexed Detection of Antimicrobial Resistance Sequences. *Nucleic Acids Res* **2019**, *47* (14), e83–e83. https://doi.org/10.1093/nar/gkz418.

(12) Wang, B.; Wang, R.; Wang, D.; Wu, J.; Li, J.; Wang, J.; Liu, H.; Wang, Y. Cas12aVDet: A CRISPR/Cas12a-Based Platform for Rapid and Visual Nucleic Acid Detection. *Anal Chem* **2019**, *91* (19), 12156–12161. https://doi.org/10.1021/acs.analchem.9b01526.

(13) Nishimasu, H.; Ran, F. A.; Hsu, P. D.; Konermann, S.; Shehata, S. I.; Dohmae, N.; Ishitani, R.; Zhang, F.; Nureki, O. Crystal Structure of Cas9 in Complex with Guide RNA and Target DNA. *Cell* **2014**, *156* (5), 935–949. https://doi.org/10.1016/j.cell.2014.02.001.

(14) Anders, C.; Niewoehner, O.; Duerst, A.; Jinek, M. Structural Basis of PAM-Dependent Target DNA Recognition by the Cas9 Endonuclease. *Nature* **2014**, *513* (7519), 569–573. https://doi.org/10.1038/nature13579.

(15) Jinek, M.; Jiang, F.; Taylor, D. W.; Sternberg, S. H.; Kaya, E.; Ma, E.; Anders, C.; Hauer, M.; Zhou, K.; Lin, S.; Kaplan, M.; Iavarone, A. T.; Charpentier, E.; Nogales, E.; Doudna, J. A. Structures of Cas9 Endonucleases Reveal RNA-Mediated Conformational Activation. *Science (1979)* **2014**, *343* (6176). https://doi.org/10.1126/science.1247997.

(16) Liu, Y.; Pinto, F.; Wan, X.; Yang, Z.; Peng, S.; Li, M.; Cooper, J. M.; Xie, Z.; French, C. E.; Wang, B. Reprogrammed TracrRNAs Enable Repurposing of RNAs as CrRNAs and Sequence-Specific RNA Biosensors. *Nat Commun* **2022**, *13* (1), 1937. https://doi.org/10.1038/s41467-022-29604-x.

(17) Yang, B.; Jin, S.; Guo, S.; Park, Y.; Chen, L.; Zhao, B.; Jung, Y. M. Recent Development of SERS Technology: Semiconductor-Based Study. *ACS Omega* **2019**, *4* (23), 20101–20108. https://doi.org/10.1021/acsomega.9b03154.

(18) Langer, J.; Jimenez de Aberasturi, D.; Aizpurua, J.; Alvarez-Puebla, R. A.; Auguié, B.; Baumberg, J. J.; Bazan, G. C.; Bell, S. E. J.; Boisen, A.; Brolo, A. G.; Choo, J.; Cialla-May, D.; Deckert, V.; Fabris, L.; Faulds, K.; García de Abajo, F. J.; Goodacre, R.; Graham, D.; Haes, A. J.; Haynes, C. L.; Huck, C.; Itoh, T.; Käll, M.; Kneipp, J.; Kotov, N. A.; Kuang, H.; Le Ru, E. C.; Lee, H. K.; Li, J.-F.; Ling, X. Y.; Maier, S. A.; Mayerhöfer, T.; Moskovits, M.; Murakoshi, K.; Nam, J.-M.; Nie, S.; Ozaki, Y.; Pastoriza-Santos, I.; Perez-Juste, J.; Popp, J.; Pucci, A.; Reich, S.; Ren, B.; Schatz, G. C.; Shegai, T.; Schlücker, S.; Tay, L.-L.; Thomas, K. G.; Tian, Z.-Q.; Van Duyne, R. P.; Vo-Dinh, T.; Wang, Y.; Willets, K. A.; Xu, C.; Xu, H.; Xu, Y.; Yamamoto, Y. S.; Zhao, B.; Liz-Marzán, L. M. Present and Future of Surface-Enhanced Raman Scattering. *ACS Nano* **2020**, *14* (1), 28–117. https://doi.org/10.1021/acsnano.9b04224.

(19) Lin, C.; Li, Y.; Peng, Y.; Zhao, S.; Xu, M.; Zhang, L.; Huang, Z.; Shi, J.; Yang, Y. Recent Development of Surface-Enhanced Raman Scattering for Biosensing. *J Nanobiotechnology* **2023**, *21* (1), 149. https://doi.org/10.1186/s12951-023-01890-7.

(20) Qian, S.; Chen, Y.; Xu, X.; Peng, C.; Wang, X.; Wu, H.; Liu, Y.; Zhong, X.; Xu, J.; Wu, J. Advances in Amplification-Free Detection of Nucleic Acid: CRISPR/Cas System as a Powerful Tool. *Anal Biochem* **2022**, *643*, 114593. https://doi.org/10.1016/j.ab.2022.114593.

(21) Nikaido, H. Multidrug Resistance in Bacteria. *Annu Rev Biochem* **2009**, *78* (1), 119–146. https://doi.org/10.1146/annurev.biochem.78.082907.145923.

(22) Kim, H.; Lee, S.; Seo, H. W.; Kang, B.; Moon, J.; Lee, K. G.; Yong, D.; Kang, H.; Jung, J.; Lim, E.-K.; Jeong, J.; Park, H. G.; Ryu, C.-M.; Kang, T. Clustered Regularly Interspaced Short Palindromic Repeats-Mediated Surface-Enhanced Raman Scattering Assay for

Multidrug-Resistant Bacteria. *ACS Nano* **2020**, *14* (12), 17241–17253. https://doi.org/10.1021/acsnano.0c07264.

(23) Hajian, R.; Balderston, S.; Tran, T.; deBoer, T.; Etienne, J.; Sandhu, M.; Wauford, N. A.; Chung, J.-Y.; Nokes, J.; Athaiya, M.; Paredes, J.; Peytavi, R.; Goldsmith, B.; Murthy, N.; Conboy, I. M.; Aran, K. Detection of Unamplified Target Genes via CRISPR–Cas9 Immobilized on a Graphene Field-Effect Transistor. *Nat Biomed Eng* **2019**, *3* (6), 427–437. https://doi.org/10.1038/s41551-019-0371-x.

(24) Balderston, S.; Taulbee, J. J.; Celaya, E.; Fung, K.; Jiao, A.; Smith, K.; Hajian, R.; Gasiunas, G.; Kutanovas, S.; Kim, D.; Parkinson, J.; Dickerson, K.; Ripoll, J.-J.; Peytavi, R.; Lu, H.-W.; Barron, F.; Goldsmith, B. R.; Collins, P. G.; Conboy, I. M.; Siksnys, V.; Aran, K. Discrimination of Single-Point Mutations in Unamplified Genomic DNA via Cas9 Immobilized on a Graphene Field-Effect Transistor. *Nat Biomed Eng* **2021**, *5* (7), 713–725. https://doi.org/10.1038/s41551-021-00706-z.

(25) Arizti-Sanz, J.; Freije, C. A.; Stanton, A. C.; Petros, B. A.; Boehm, C. K.; Siddiqui, S.; Shaw, B. M.; Adams, G.; Kosoko-Thoroddsen, T.-S. F.; Kemball, M. E.; Uwanibe, J. N.; Ajogbasile, F. V.; Eromon, P. E.; Gross, R.; Wronka, L.; Caviness, K.; Hensley, L. E.; Bergman, N. H.; MacInnis, B. L.; Happi, C. T.; Lemieux, J. E.; Sabeti, P. C.; Myhrvold, C. Streamlined Inactivation, Amplification, and Cas13-Based Detection of SARS-CoV-2. *Nat Commun* **2020**, *11* (1), 5921. https://doi.org/10.1038/s41467-020-19097-x.

(26) Arizti-Sanz, J.; Bradley, A.; Zhang, Y. B.; Boehm, C. K.; Freije, C. A.; Grunberg, M. E.; Kosoko-Thoroddsen, T.-S. F.; Welch, N. L.; Pillai, P. P.; Mantena, S.; Kim, G.; Uwanibe, J. N.; John, O. G.; Eromon, P. E.; Kocher, G.; Gross, R.; Lee, J. S.; Hensley, L. E.; MacInnis, B. L.; Johnson, J.; Springer, M.; Happi, C. T.; Sabeti, P. C.; Myhrvold, C. Simplified Cas13-Based Assays for the Fast Identification of SARS-CoV-2 and Its Variants. *Nat Biomed Eng* **2022**, *6* (8), 932–943. https://doi.org/10.1038/s41551-022-00889-z.

(27) Ji, C.; Shao, J. Shine: A Novel Strategy to Extract Specific, Sensitive and Well-Conserved Biomarkers from Massive Microbial Genomic Datasets. *BMC Bioinformatics* **2023**, *24* (1), 128. https://doi.org/10.1186/s12859-023-05195-2.

(28) Fozouni, P.; Son, S.; Díaz de León Derby, M.; Knott, G. J.; Gray, C. N.; D'Ambrosio, M. v.; Zhao, C.; Switz, N. A.; Kumar, G. R.; Stephens, S. I.; Boehm, D.; Tsou, C.-L.; Shu, J.; Bhuiya, A.; Armstrong, M.; Harris, A. R.; Chen, P.-Y.; Osterloh, J. M.; Meyer-Franke, A.;

Joehnk, B.; Walcott, K.; Sil, A.; Langelier, C.; Pollard, K. S.; Crawford, E. D.; Puschnik, A. S.; Phelps, M.; Kistler, A.; DeRisi, J. L.; Doudna, J. A.; Fletcher, D. A.; Ott, M. Amplification-Free Detection of SARS-CoV-2 with CRISPR-Cas13a and Mobile Phone Microscopy. *Cell* **2021**, *184* (2), 323-333.e9. https://doi.org/10.1016/j.cell.2020.12.001.

(29) Breslauer, D. N.; Maamari, R. N.; Switz, N. A.; Lam, W. A.; Fletcher, D. A. Mobile Phone Based Clinical Microscopy for Global Health Applications. *PLoS One* **2009**, *4* (7), e6320. https://doi.org/10.1371/journal.pone.0006320.

(30) Hou, T.; Zeng, W.; Yang, M.; Chen, W.; Ren, L.; Ai, J.; Wu, J.; Liao, Y.; Gou, X.; Li, Y.; Wang, X.; Su, H.; Gu, B.; Wang, J.; Xu, T. Development and Evaluation of a Rapid CRISPR-Based Diagnostic for COVID-19. *PLoS Pathog* **2020**, *16* (8), e1008705. https://doi.org/10.1371/journal.ppat.1008705.

(31) Fung, T. S.; Liu, D. X. Human Coronavirus: Host-Pathogen Interaction. *Annu Rev Microbiol* **2019**, *73* (1), 529–557. https://doi.org/10.1146/annurev-micro-020518-115759.

(32) Wang, D.; Wang, X.; Ye, F.; Zou, J.; Qu, J.; Jiang, X. An Integrated Amplification-Free Digital CRISPR/Cas-Assisted Assay for Single Molecule Detection of RNA. *ACS Nano* **2023**, *17* (8), 7250–7256. https://doi.org/10.1021/acsnano.2c10143.

(33) Kashefi-Kheyrabadi, L.; Nguyen, H. V.; Go, A.; Lee, M.-H. Ultrasensitive and Amplification-Free Detection of SARS-CoV-2 RNA Using an Electrochemical Biosensor Powered by CRISPR/Cas13a. *Bioelectrochemistry* **2023**, *150*, 108364. https://doi.org/10.1016/j.bioelechem.2023.108364.

(34) Heo, W.; Lee, K.; Park, S.; Hyun, K.-A.; Jung, H.-I. Electrochemical Biosensor for Nucleic Acid Amplification-Free and Sensitive Detection of Severe Acute Respiratory Syndrome Coronavirus 2 (SARS-CoV-2) RNA via CRISPR/Cas13a Trans-Cleavage Reaction. *Biosens Bioelectron* **2022**, *201*, 113960. https://doi.org/10.1016/j.bios.2021.113960.

(35) Hang, X.-M.; Liu, P.-F.; Tian, S.; Wang, H.-Y.; Zhao, K.-R.; Wang, L. Rapid and Sensitive Detection of Ebola RNA in an Unamplified Sample Based on CRISPR-Cas13a and DNA Roller Machine. *Biosens Bioelectron* **2022**, *211*, 114393. https://doi.org/10.1016/j.bios.2022.114393.

(36) Cheng, G. Circulating MiRNAs: Roles in Cancer Diagnosis, Prognosis and Therapy. *Adv Drug Deliv Rev* **2015**, *81*, 75–93. https://doi.org/10.1016/j.addr.2014.09.001.

(37) Bruch, R.; Baaske, J.; Chatelle, C.; Meirich, M.; Madlener, S.; Weber, W.; Dincer, C.; Urban, G. A. CRISPR/Cas13a-Powered Electrochemical Microfluidic Biosensor for Nucleic Acid Amplification-Free MiRNA Diagnostics. *Advanced Materials* **2019**, *31* (51), 1905311. https://doi.org/10.1002/adma.201905311.

(38) Xu, Y.; Wang, C.; Liu, G.; Zhao, X.; Qian, Q.; Li, S.; Mi, X. Tetrahedral DNA Framework Based CRISPR Electrochemical Biosensor for Amplification-Free MiRNA Detection. *Biosens Bioelectron* **2022**, *217*, 114671. https://doi.org/10.1016/j.bios.2022.114671.

(39) Liu, L.; Wang, Z.; Wang, Y.; Luan, J.; Morrissey, J. J.; Naik, R. R.; Singamaneni, S. Plasmonically Enhanced CRISPR/Cas13a-Based Bioassay for Amplification-Free Detection of Cancer-Associated RNA. *Adv Healthc Mater* **2021**, *10* (20), 2100956. https://doi.org/10.1002/adhm.202100956.

(40) Volik, S.; Alcaide, M.; Morin, R. D.; Collins, C. Cell-Free DNA (CfDNA): Clinical Significance and Utility in Cancer Shaped By Emerging Technologies. *Molecular Cancer Research* **2016**, *14* (10), 898–908. https://doi.org/10.1158/1541-7786.MCR-16-0044.

(41) Choi, J.-H.; Lim, J.; Shin, M.; Paek, S.-H.; Choi, J.-W. CRISPR-Cas12a-Based Nucleic Acid Amplification-Free DNA Biosensor via Au Nanoparticle-Assisted Metal-Enhanced Fluorescence and Colorimetric Analysis. *Nano Lett* **2021**, *21* (1), 693–699. https://doi.org/10.1021/acs.nanolett.0c04303.

(42) Yue, H.; Shu, B.; Tian, T.; Xiong, E.; Huang, M.; Zhu, D.; Sun, J.; Liu, Q.; Wang, S.; Li, Y.; Zhou, X. Droplet Cas12a Assay Enables DNA Quantification from Unamplified Samples at the Single-Molecule Level. *Nano Lett* **2021**, *21* (11), 4643–4653. https://doi.org/10.1021/acs.nanolett.1c00715.

(43) Wang, Y.; Luo, J.; Liu, J.; Sun, S.; Xiong, Y.; Ma, Y.; Yan, S.; Yang, Y.; Yin, H.; Cai, X. Label-Free Microfluidic Paper-Based Electrochemical Aptasensor for Ultrasensitive and Simultaneous Multiplexed Detection of Cancer Biomarkers. *Biosens Bioelectron* **2019**, *136*, 84–90. https://doi.org/10.1016/j.bios.2019.04.032.

(44) Xu, Y.; Zhang, X.; Luan, C.; Wang, H.; Chen, B.; Zhao, Y. Hybrid Hydrogel Photonic Barcodes for Multiplex Detection of Tumor Markers. *Biosens Bioelectron* **2017**, *87*, 264–270. https://doi.org/10.1016/j.bios.2016.08.063.

(45) Chen, H.; Chen, C.; Bai, S.; Gao, Y.; Metcalfe, G.; Cheng, W.; Zhu, Y. Multiplexed Detection of Cancer Biomarkers Using a Microfluidic Platform Integrating Single Bead

Trapping and Acoustic Mixing Techniques. *Nanoscale* **2018**, *10* (43), 20196–20206. https://doi.org/10.1039/C8NR06367B.

(46) Gao, W.; Wang, W.; Yao, S.; Wu, S.; Zhang, H.; Zhang, J.; Jing, F.; Mao, H.; Jin, Q.; Cong, H.; Jia, C.; Zhang, G.; Zhao, J. Highly Sensitive Detection of Multiple Tumor Markers for Lung Cancer Using Gold Nanoparticle Probes and Microarrays. *Anal Chim Acta* **2017**, *958*, 77–84. https://doi.org/10.1016/j.aca.2016.12.016.

(47) Chen, F.; Hu, Q.; Li, H.; Xie, Y.; Xiu, L.; Zhang, Y.; Guo, X.; Yin, K. Multiplex Detection of Infectious Diseases on Microfluidic Platforms. *Biosensors (Basel)* **2023**, *13* (3), 410. https://doi.org/10.3390/bios13030410.

(48) Mahony, J. B.; Blackhouse, G.; Babwah, J.; Smieja, M.; Buracond, S.; Chong, S.; Ciccotelli, W.; O'Shea, T.; Alnakhli, D.; Griffiths-Turner, M.; Goeree, R. Cost Analysis of Multiplex PCR Testing for Diagnosing Respiratory Virus Infections. *J Clin Microbiol* **2009**, *47* (9), 2812–2817. https://doi.org/10.1128/JCM.00556-09.

(49) Huang, L.; Tian, S.; Zhao, W.; Liu, K.; Ma, X.; Guo, J. Multiplexed Detection of Biomarkers in Lateral-Flow Immunoassays. *Analyst* **2020**, *145* (8), 2828–2840. https://doi.org/10.1039/C9AN02485A.

(50) Hori, S. S.; Gambhir, S. S. Mathematical Model Identifies Blood Biomarker–Based Early Cancer Detection Strategies and Limitations. *Sci Transl Med* **2011**, *3* (109). https://doi.org/10.1126/scitranslmed.3003110.

(51) Kwong, G. A.; von Maltzahn, G.; Murugappan, G.; Abudayyeh, O.; Mo, S.; Papayannopoulos, I. A.; Sverdlov, D. Y.; Liu, S. B.; Warren, A. D.; Popov, Y.; Schuppan, D.; Bhatia, S. N. Mass-Encoded Synthetic Biomarkers for Multiplexed Urinary Monitoring of Disease. *Nat Biotechnol* **2013**, *31* (1), 63–70. https://doi.org/10.1038/nbt.2464.

(52) Kwong, G. A.; Ghosh, S.; Gamboa, L.; Patriotis, C.; Srivastava, S.; Bhatia, S. N. Synthetic Biomarkers: A Twenty-First Century Path to Early Cancer Detection. *Nat Rev Cancer* **2021**, *21* (10), 655–668. https://doi.org/10.1038/s41568-021-00389-3.

(53) Dahlman, J. E.; Kauffman, K. J.; Xing, Y.; Shaw, T. E.; Mir, F. F.; Dlott, C. C.; Langer, R.; Anderson, D. G.; Wang, E. T. Barcoded Nanoparticles for High Throughput in Vivo Discovery of Targeted Therapeutics. *Proceedings of the National Academy of Sciences* **2017**, *114* (8), 2060–2065. https://doi.org/10.1073/pnas.1620874114.

(54) Mamanova, L.; Coffey, A. J.; Scott, C. E.; Kozarewa, I.; Turner, E. H.; Kumar, A.; Howard, E.; Shendure, J.; Turner, D. J. Target-Enrichment Strategies for next-Generation Sequencing. *Nat Methods* **2010**, *7* (2), 111–118. https://doi.org/10.1038/nmeth.1419.

(55) Fan, H.; Chu, J.-Y. A Brief Review of Short Tandem Repeat Mutation. *Genomics Proteomics Bioinformatics* **2007**, *5* (1), 7–14. https://doi.org/10.1016/S1672-0229(07)60009-6.

(56) Chintalaphani, S. R.; Pineda, S. S.; Deveson, I. W.; Kumar, K. R. An Update on the Neurological Short Tandem Repeat Expansion Disorders and the Emergence of Long-Read Sequencing Diagnostics. *Acta Neuropathol Commun* **2021**, *9* (1), 98. https://doi.org/10.1186/s40478-021-01201-x.

(57) Rajan-Babu, I.-S.; Peng, J. J.; Chiu, R.; Birch, P.; Couse, M.; Guimond, C.; Lehman, A.; Mwenifumbo, J.; van Karnebeek, C.; Friedman, J.; Adam, S.; Souich, C. Du; Elliott, A.; Lehman, A.; Mwenifumbo, J.; Nelson, T.; van Karnebeek, C.; Friedman, J.; Li, C.; Mohajeri, A.; Dolzhenko, E.; Eberle, M. A.; Birol, I.; Friedman, J. M. Genome-Wide Sequencing as a First-Tier Screening Test for Short Tandem Repeat Expansions. *Genome Med* **2021**, *13* (1), 126. https://doi.org/10.1186/s13073-021-00932-9.

(58) Zhou, D.; Tan, L.; Li, J.; Liu, T.; Hu, Y.; Li, Y.; Kawamoto, S.; Liu, C.; Guo, S.; Wang, A. Identification of Homologous Recombination Events in Mouse Embryonic Stem Cells Using Southern Blotting and Polymerase Chain Reaction. *Journal of Visualized Experiments* **2018**, No. 141. https://doi.org/10.3791/58467.

(59) WILCZYNSKI, S. P. Molecular Biology. In *Modern Surgical Pathology*; Elsevier, 2009; pp 85–120. https://doi.org/10.1016/B978-1-4160-3966-2.00006-0.

(60) Chintalaphani, S. R.; Pineda, S. S.; Deveson, I. W.; Kumar, K. R. An Update on the Neurological Short Tandem Repeat Expansion Disorders and the Emergence of Long-Read Sequencing Diagnostics. *Acta Neuropathol Commun* **2021**, *9* (1), 98. https://doi.org/10.1186/s40478-021-01201-x.

(61) Stevens, A. J.; Taylor, M. G.; Pearce, F. G.; Kennedy, M. A. Allelic Dropout During Polymerase Chain Reaction Due to G-Quadruplex Structures and DNA Methylation Is Widespread at Imprinted Human Loci. *G3 Genes|Genomes|Genetics* **2017**, *7* (3), 1019–1025. https://doi.org/10.1534/g3.116.038687.

(62) Shin, G.; Grimes, S. M.; Lee, H.; Lau, B. T.; Xia, L. C.; Ji, H. P. CRISPR–Cas9-Targeted Fragmentation and Selective Sequencing Enable Massively Parallel Microsatellite Analysis. *Nat Commun* **2017**, *8* (1), 14291. https://doi.org/10.1038/ncomms14291.

(63) Hafford-Tear, N. J.; Tsai, Y.-C.; Sadan, A. N.; Sanchez-Pintado, B.; Zarouchlioti, C.; Maher, G. J.; Liskova, P.; Tuft, S. J.; Hardcastle, A. J.; Clark, T. A.; Davidson, A. E. CRISPR/Cas9-Targeted Enrichment and Long-Read Sequencing of the Fuchs Endothelial Corneal Dystrophy–Associated TCF4 Triplet Repeat. *Genetics in Medicine* **2019**, *21* (9), 2092–2102. https://doi.org/10.1038/s41436-019-0453-x.

(64) Wieben, E. D.; Aleff, R. A.; Basu, S.; Sarangi, V.; Bowman, B.; McLaughlin, I. J.; Mills, J. R.; Butz, M. L.; Highsmith, E. W.; Ida, C. M.; Ekholm, J. M.; Baratz, K. H.; Fautsch, M. P. Amplification-Free Long-Read Sequencing of TCF4 Expanded Trinucleotide Repeats in Fuchs Endothelial Corneal Dystrophy. *PLoS One* **2019**, *14* (7), e0219446. https://doi.org/10.1371/journal.pone.0219446.

(65) Malekshoar, M.; Azimi, S. A.; Kaki, A.; Mousazadeh, L.; Motaei, J.; Vatankhah, M. CRISPR-Cas9 Targeted Enrichment and Next-Generation Sequencing for Mutation Detection. *The Journal of Molecular Diagnostics* **2023**, *25* (5), 249–262. https://doi.org/10.1016/j.jmoldx.2023.01.010.

(66) Acquah, C.; Agyei, D.; Obeng, E. M.; Pan, S.; Tan, K. X.; Danquah, M. K. Aptamers: An Emerging Class of Bioaffinity Ligands in Bioactive Peptide Applications. *Crit Rev Food Sci Nutr* **2020**, *60* (7), 1195–1206. https://doi.org/10.1080/10408398.2018.1564234.

(67) Yee, B. J.; Shafiqah, N. F.; Mohd-Naim, N. F.; Ahmed, M. U. A CRISPR/Cas12a-Based Fluorescence Aptasensor for the Rapid and Sensitive Detection of Ampicillin. *Int J Biol Macromol* **2023**, *242*, 125211. https://doi.org/10.1016/j.ijbiomac.2023.125211.

(68) Wei, Y.; Tao, Z.; Wan, L.; Zong, C.; Wu, J.; Tan, X.; Wang, B.; Guo, Z.; Zhang, L.; Yuan, H.; Wang, P.; Yang, Z.; Wan, Y. Aptamer-Based Cas14a1 Biosensor for Amplification-Free Live Pathogenic Detection. *Biosens Bioelectron* **2022**, *211*, 114282. https://doi.org/10.1016/j.bios.2022.114282.

(69) Duan, M.; Li, B.; Zhao, Y.; Liu, Y.; Liu, Y.; Dai, R.; Li, X.; Jia, F. A CRISPR/Cas12a-Mediated, DNA Extraction and Amplification-Free, Highly Direct and Rapid Biosensor for Salmonella Typhimurium. *Biosens Bioelectron* **2023**, *219*, 114823. https://doi.org/10.1016/j.bios.2022.114823.

(70) Kong, M.; Shin, J. H.; Heu, S.; Park, J.-K.; Ryu, S. Lateral Flow Assay-Based Bacterial Detection Using Engineered Cell Wall Binding Domains of a Phage Endolysin. *Biosens Bioelectron* **2017**, *96*, 173–177. https://doi.org/10.1016/j.bios.2017.05.010.

(71) Yao, Y.; Li, S.; Cao, J.; Liu, W.; Fan, K.; Xiang, W.; Yang, K.; Kong, D.; Wang, W. Development of Small Molecule Biosensors by Coupling the Recognition of the Bacterial Allosteric Transcription Factor with Isothermal Strand Displacement Amplification. *Chemical Communications* **2018**, *54* (38), 4774–4777. https://doi.org/10.1039/C8CC01764F.

(72) Li, S.; Zhou, L.; Yao, Y.; Fan, K.; Li, Z.; Zhang, L.; Wang, W.; Yang, K. A Platform for the Development of Novel Biosensors by Configuring Allosteric Transcription Factor Recognition with Amplified Luminescent Proximity Homogeneous Assays. *Chemical Communications* **2017**, *53* (1), 99–102. https://doi.org/10.1039/C6CC07244E.

(73) Zhao, X.; Zhang, W.; Qiu, X.; Mei, Q.; Luo, Y.; Fu, W. Rapid and Sensitive Exosome Detection with CRISPR/Cas12a. *Anal Bioanal Chem* **2020**, *412* (3), 601–609. https://doi.org/10.1007/s00216-019-02211-4.

(74) Liang, M.; Li, Z.; Wang, W.; Liu, J.; Liu, L.; Zhu, G.; Karthik, L.; Wang, M.; Wang, K.-F.; Wang, Z.; Yu, J.; Shuai, Y.; Yu, J.; Zhang, L.; Yang, Z.; Li, C.; Zhang, Q.; Shi, T.; Zhou, L.; Xie, F.; Dai, H.; Liu, X.; Zhang, J.; Liu, G.; Zhuo, Y.; Zhang, B.; Liu, C.; Li, S.; Xia, X.; Tong, Y.; Liu, Y.; Alterovitz, G.; Tan, G.-Y.; Zhang, L.-X. A CRISPR-Cas12a-Derived Biosensing Platform for the Highly Sensitive Detection of Diverse Small Molecules. *Nat Commun* **2019**, *10* (1), 3672. https://doi.org/10.1038/s41467-019-11648-1.

(75) Iwasaki, R. S.; Batey, R. T. SPRINT: A Cas13a-Based Platform for Detection of Small Molecules. *Nucleic Acids Res* **2020**, *48* (17), e101–e101. https://doi.org/10.1093/nar/gkaa673.

(76) Mahas, A.; Wang, Q.; Marsic, T.; Mahfouz, M. M. Development of Cas12a-Based Cell-Free Small-Molecule Biosensors via Allosteric Regulation of CRISPR Array Expression. *Anal Chem* **2022**, *94* (11), 4617–4626. https://doi.org/10.1021/acs.analchem.1c04332.

(77) Mahas, A.; Wang, Q.; Marsic, T.; Mahfouz, M. M. Development of Cas12a-Based Cell-Free Small-Molecule Biosensors via Allosteric Regulation of CRISPR Array Expression. *Anal Chem* **2022**, *94* (11), 4617–4626. https://doi.org/10.1021/acs.analchem.1c04332.

(78) Liang, M.; Li, Z.; Wang, W.; Liu, J.; Liu, L.; Zhu, G.; Karthik, L.; Wang, M.; Wang, K.-F.; Wang, Z.; Yu, J.; Shuai, Y.; Yu, J.; Zhang, L.; Yang, Z.; Li, C.; Zhang, Q.; Shi, T.; Zhou, L.; Xie, F.; Dai, H.; Liu, X.; Zhang, J.; Liu, G.; Zhuo, Y.; Zhang, B.; Liu, C.; Li, S.; Xia, X.; Tong, Y.; Liu, Y.; Alterovitz, G.; Tan, G.-Y.; Zhang, L.-X. A CRISPR-Cas12a-Derived Biosensing Platform for the Highly Sensitive Detection of Diverse Small Molecules. *Nat Commun* **2019**, *10* (1), 3672. https://doi.org/10.1038/s41467-019-11648-1.

(79) Wang, Y.; Peng, Y.; Li, S.; Han, D.; Ren, S.; Qin, K.; Zhou, H.; Han, T.; Gao, Z. The Development of a Fluorescence/Colorimetric Biosensor Based on the Cleavage Activity of CRISPR-Cas12a for the Detection of Non-Nucleic Acid Targets. *J Hazard Mater* **2023**, *449*, 131044. https://doi.org/10.1016/j.jhazmat.2023.131044.

(80) Najjar, D.; Rainbow, J.; Sharma Timilsina, S.; Jolly, P.; de Puig, H.; Yafia, M.; Durr, N.; Sallum, H.; Alter, G.; Li, J. Z.; Yu, X. G.; Walt, D. R.; Paradiso, J. A.; Estrela, P.; Collins, J. J.; Ingber, D. E. A Lab-on-a-Chip for the Concurrent Electrochemical Detection of SARS-CoV-2 RNA and Anti-SARS-CoV-2 Antibodies in Saliva and Plasma. *Nat Biomed Eng* **2022**, *6* (8), 968–978. https://doi.org/10.1038/s41551-022-00919-w.

(81) Katzmeier, F.; Aufinger, L.; Dupin, A.; Quintero, J.; Lenz, M.; Bauer, L.; Klumpe, S.; Sherpa, D.; Dürr, B.; Honemann, M.; Styazhkin, I.; Simmel, F. C.; Heymann, M. A Low-Cost Fluorescence Reader for in Vitro Transcription and Nucleic Acid Detection with Cas13a. *PLoS One* **2019**, *14* (12), e0220091. https://doi.org/10.1371/journal.pone.0220091.

(82) Xie, S.; Ji, Z.; Suo, T.; Li, B.; Zhang, X. Advancing Sensing Technology with CRISPR: From the Detection of Nucleic Acids to a Broad Range of Analytes – A Review. *Anal Chim Acta* **2021**, *1185*, 338848. https://doi.org/10.1016/j.aca.2021.338848.

(83) Chen, K.; Shen, Z.; Wang, G.; Gu, W.; Zhao, S.; Lin, Z.; Liu, W.; Cai, Y.; Mushtaq, G.; Jia, J.; Wan, C. (Craig); Yan, T. Research Progress of CRISPR-Based Biosensors and Bioassays for Molecular Diagnosis. *Front Bioeng Biotechnol* **2022**, *10*. https://doi.org/10.3389/fbioe.2022.986233.

(84) Deng, Y.; Jiang, H.; Li, X.; Lv, X. Recent Advances in Sensitivity Enhancement for Lateral Flow Assay. *Microchimica Acta* **2021**, *188* (11), 379. https://doi.org/10.1007/s00604-021-05037-z.

(85) Kasetsirikul, S.; Shiddiky, M. J. A.; Nguyen, N.-T. Challenges and Perspectives in the Development of Paper-Based Lateral Flow Assays. *Microfluid Nanofluidics* **2020**, *24* (2), 17. https://doi.org/10.1007/s10404-020-2321-z.

(86) Shao, N.; Han, X.; Song, Y.; Zhang, P.; Qin, L. CRISPR-Cas12a Coupled with Platinum Nanoreporter for Visual Quantification of SNVs on a Volumetric Bar-Chart Chip. *Anal Chem* **2019**, *91* (19), 12384–12391. https://doi.org/10.1021/acs.analchem.9b02925.

(87) Dai, Y.; Somoza, R. A.; Wang, L.; Welter, J. F.; Li, Y.; Caplan, A. I.; Liu, C. C. Exploring the Trans-Cleavage Activity of CRISPR-Cas12a (Cpf1) for the Development of a Universal Electrochemical Biosensor. *Angewandte Chemie International Edition* **2019**, *58* (48), 17399–17405. https://doi.org/10.1002/anie.201910772.

(88) Cao, Y.; Zheng, Z.; Monbouquette, H. G. Nucleic Acid Amplification-Free Detection of DNA and RNA at Ultralow Concentration. *Curr Opin Biotechnol* **2021**, *71*, 145–150. https://doi.org/10.1016/j.copbio.2021.07.022.

(89) Ding, X.; Yin, K.; Li, Z.; Lalla, R. v.; Ballesteros, E.; Sfeir, M. M.; Liu, C. Ultrasensitive and Visual Detection of SARS-CoV-2 Using All-in-One Dual CRISPR-Cas12a Assay. *Nat Commun* **2020**, *11* (1), 4711. https://doi.org/10.1038/s41467-020-18575-6.

(90) Arizti-Sanz, J.; Freije, C. A.; Stanton, A. C.; Petros, B. A.; Boehm, C. K.; Siddiqui, S.; Shaw, B. M.; Adams, G.; Kosoko-Thoroddsen, T.-S. F.; Kemball, M. E.; Uwanibe, J. N.; Ajogbasile, F. v.; Eromon, P. E.; Gross, R.; Wronka, L.; Caviness, K.; Hensley, L. E.; Bergman, N. H.; MacInnis, B. L.; Happi, C. T.; Lemieux, J. E.; Sabeti, P. C.; Myhrvold, C. Streamlined Inactivation, Amplification, and Cas13-Based Detection of SARS-CoV-2. *Nat Commun* **2020**, *11* (1), 5921. https://doi.org/10.1038/s41467-020-19097-x.

(91) Yudin Kharismasari, C.; Irkham; Zein, M. I. H. L.; Hardianto, A.; Nur Zakiyyah, S.; Umar Ibrahim, A.; Ozsoz, M.; Wahyuni Hartati, Y. CRISPR/Cas12-Based Electrochemical Biosensors for Clinical Diagnostic and Food Monitoring. *Bioelectrochemistry* **2024**, *155*, 108600. https://doi.org/10.1016/j.bioelechem.2023.108600.

(92) Wu, C.; Chen, Z.; Li, C.; Hao, Y.; Tang, Y.; Yuan, Y.; Chai, L.; Fan, T.; Yu, J.; Ma, X.; Al-Hartomy, O. A.; Wageh, S.; Al-Sehemi, A. G.; Luo, Z.; He, Y.; Li, J.; Xie, Z.; Zhang, H. CRISPR-Cas12a-Empowered Electrochemical Biosensor for Rapid and Ultrasensitive Detection of SARS-CoV-2 Delta Variant. *Nanomicro Lett* **2022**, *14* (1), 159. https://doi.org/10.1007/s40820-022-00888-4.

(93) Mao, Z.; Chen, R.; Wang, X.; Zhou, Z.; Peng, Y.; Li, S.; Han, D.; Li, S.; Wang, Y.; Han, T.; Liang, J.; Ren, S.; Gao, Z. CRISPR/Cas12a-Based Technology: A Powerful Tool for Biosensing in Food Safety. *Trends Food Sci Technol* **2022**, *122*, 211–222. https://doi.org/10.1016/j.tifs.2022.02.030.

(94) Liu, S.; Xie, T.; Huang, Z.; Pei, X.; Li, S.; He, Y.; Tong, Y.; Liu, G. Systematically Investigating the Fluorescent Signal Readout of CRISPR-Cas12a for Highly Sensitive SARS-CoV-2 Detection. *Sens Actuators B Chem* **2022**, *373*, 132746. https://doi.org/10.1016/j.snb.2022.132746.

(95) Allen, D.; Rosenberg, M.; Hendel, A. Using Synthetically Engineered Guide RNAs to Enhance CRISPR Genome Editing Systems in Mammalian Cells. *Front Genome Ed* **2021**, *2*. https://doi.org/10.3389/fgeed.2020.617910.

(96) Zeng, M.; Ke, Y.; Zhuang, Z.; Qin, C.; Li, L. Y.; Sheng, G.; Li, Z.; Meng, H.; Ding, X. Harnessing Multiplex CrRNA in the CRISPR/Cas12a System Enables an Amplification-Free DNA Diagnostic Platform for ASFV Detection. *Anal Chem* **2022**, *94* (30), 10805–10812. https://doi.org/10.1021/acs.analchem.2c01588.

(97) Chen, Y.; Yang, T.; Qian, S.; Peng, C.; Wang, X.; Wang, T.; Che, Y.; Ji, F.; Wu, J.; Xu, J. Multiple CrRNAs-Assisted CRISPR/Cas12a Assay Targeting Cytochrome b Gene for Amplification-Free Detection of Meat Adulteration. *Anal Chim Acta* **2022**, *1231*, 340417. https://doi.org/10.1016/j.aca.2022.340417.

(98) Ramachandran, A.; Santiago, J. G. CRISPR Enzyme Kinetics for Molecular Diagnostics. *Anal Chem* **2021**, *93* (20), 7456–7464. https://doi.org/10.1021/acs.analchem.1c00525.

(99) Liu, X.; Kang, X.; Lei, C.; Ren, W.; Liu, C. Programming the *Trans* -Cleavage Activity of CRISPR-Cas13a by Single-Strand DNA Blocker and Its Biosensing Application. *Anal Chem* **2022**, *94* (9), 3987–3996. https://doi.org/10.1021/acs.analchem.1c05124.

(100) Deng, F.; Li, Y.; Li, B.; Goldys, E. M. Increasing Trans-Cleavage Catalytic Efficiency of Cas12a and Cas13a with Chemical Enhancers: Application to Amplified Nucleic Acid Detection. *Sens Actuators B Chem* **2022**, *373*, 132767. https://doi.org/10.1016/j.snb.2022.132767.

(101) Lv, H.; Wang, J.; Zhang, J.; Chen, Y.; Yin, L.; Jin, D.; Gu, D.; Zhao, H.; Xu, Y.; Wang, J. Definition of CRISPR Cas12a Trans-Cleavage Units to Facilitate CRISPR Diagnostics. *Front Microbiol* **2021**, *12*. https://doi.org/10.3389/fmicb.2021.766464.

(102) Nguyen, L. T.; Smith, B. M.; Jain, P. K. Enhancement of Trans-Cleavage Activity of Cas12a with Engineered CrRNA Enables Amplified Nucleic Acid Detection. *Nat Commun* **2020**, *11* (1), 4906. https://doi.org/10.1038/s41467-020-18615-1.

(103) Feng, W.; Zhang, H.; Le, X. C. Signal Amplification by the *Trans* -Cleavage Activity of CRISPR-Cas Systems: Kinetics and Performance. *Anal Chem* **2023**, *95* (1), 206–217. https://doi.org/10.1021/acs.analchem.2c04555.

(104) Curtin, K.; Fike, B. J.; Binkley, B.; Godary, T.; Li, P. Recent Advances in Digital Biosensing Technology. *Biosensors (Basel)* **2022**, *12* (9), 673. https://doi.org/10.3390/bios12090673.
